# Multifunctional Oxidized Dextran–Metformin as a Tissue‐Adhesive Hydrogel to Prevent Postoperative Peritoneal Adhesions in Patients with Metabolic Syndrome

**DOI:** 10.1002/advs.202303767

**Published:** 2023-10-16

**Authors:** Xi Liu, Xianwen Song, Zequn Zhang, Shutong Yang, Liang Li, Changwei Lin, Miao Chen, Chuntai Liu, Xiaorong Li, Yi Zhang, Gui Hu

**Affiliations:** ^1^ Department of Gastrointestinal Surgery The Third Xiangya Hospital of Central South University Tongzipo Road Changsha Hunan 410013 P. R. China; ^2^ Hunan Provincial Key Laboratory of Micro & Nano Materials Interface Science College of Chemistry and Chemical Engineering Central South University Changsha 410083 P. R. China; ^3^ National Engineering Research Center for Advanced Polymer Processing Technology Zhengzhou University Zhengzhou 450002 P. R. China

**Keywords:** hydrogel, metabolic syndrome, metformin, peritoneal adhesions

## Abstract

Patients with metabolic syndrome (MetS) undergoing surgery are at high risk of developing peritoneal adhesions and other severe postoperative complications. However, the single shielding function and absence of physiological activity render conventional methods less useful in preventing adhesions in patients with MetS. To address this challenge, a convenient method is introduced for developing a novel tissue‐adhesive hydrogel called oxidized dextran–metformin (ODE–ME) via Schiff base linkages. This injectable ODE–ME hydrogel exhibits excellent tissue‐adhesive properties and various physiological functions, particularly enhanced antibacterial effects. Furthermore, in vivo experiments demonstrate that the hydrogel can effectively alleviate hyperglycemia, reduce excessive inflammation, and improve fibrinolytic activity in MetS mice, thereby preventing adhesions and promoting incisional healing. The hydrogel concurrently isolates injured tissues and lowers the blood glucose levels immediately after surgery in mice. Therefore, the ODE–ME hydrogel functions as a multifunctional barrier material and has potential for preventing postoperative peritoneal adhesions in patients with MetS in clinical settings.

## Graphical abstract

Synthesis of ODE–ME hydrogel and its effect in mice with MetS. A) Schematic illustration of ODE‐ME hydrogel synthesis. B) ODE–ME hydrogel has antifouling, antibacterial, and antioxidative effects, and can prevent severe postoperative peritoneal adhesions in mice with MetS, relieve hyperglycemia, inhibit excessive inflammatory activation, and reduce fibrinolytic imbalance.

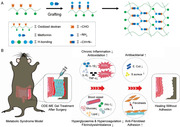

## 1 Introduction

Metabolic syndrome (MetS) is characterized by a cluster of metabolic dysregulations including obesity, hyperglycemia, dyslipidemia, and hypertension.^[^ ^1^ ^]^ Its prevalence is high worldwide, particularly in the developed countries.^[^ ^2^ ^]^ Patients with MetS have an increased risk of postoperative complications such as delayed wound healing, bacterial infections, and peritoneal adhesions.^[^ ^3^ ^]^ Peritoneal adhesions tend to be more severe in patients with MetS following abdominal surgery.^[^ ^4^ ^]^ These adhesions can lead to chronic abdominal pain, female infertility, intestinal obstruction, organ dysfunction, and increased mortality risk.^[^ ^5^ ^]^ Several factors contribute to this pathology, including prolonged operation time, severe surgical injuries resulting from obesity, chronic inflammation, reduced fibrinolytic activity, and a hypercoagulable state.^[^ ^6^ ^]^ Currently, limited studies have addressed postoperative peritoneal adhesions in patients with MetS; consequently, there is a lack of relevant animal experimental models. Furthermore, the biodegradable membranes and hydrogels commonly employed in clinical practice offer limited effectiveness in preventing adhesions, as they do not fully guard against bacterial infections or degrade rapidly in vivo. These materials fail to target the molecular mechanisms underlying postoperative peritoneal adhesions in patients with MetS.^[^ ^7^ ^]^ Despite a few accidental laboratory discoveries,^[^ ^8^ ^]^ there is an urgent need for prevention and treatment strategies for postoperative peritoneal adhesions in patients with MetS. This study aims to establish an animal model of postoperative peritoneal adhesions in the presence of MetS and introduces a novel hydrogel material designed to serve as a physical barrier and to target the molecular mechanisms responsible for postoperative peritoneal adhesions in patients with MetS.

Metformin (ME), commonly used to treat MetS‐associated hyperglycemia, is an affordable medication that reduces blood glucose and lipid levels, oxidative stress, and inflammation, promotes fibrinolysis, and inhibits fibrosis.^[^ ^9^ ^]^ In theory, it is an ideal drug for preventing postoperative intra‐abdominal adhesions in patients with MetS. However, few studies have explored the use of ME in the treatment of peritoneal adhesions after intra‐abdominal surgery. We hypothesized that an effective method for preventing postoperative peritoneal adhesions in patients with MetS is to synthesize a novel hydrogel barrier material based on ME to be directly administered into the abdominal cavity while maintaining drug concentration, safety, and stability.

This study presents a simple design, preparation, and application of a tissue‐adhesive hydrogel that combines ME grafting with oxidized dextran (ODE) via a Schiff base reaction. Molecular simulations and spectroscopic characterizations confirmed that the gelation of the ODE–ME compound resulted from multiple cross‐linking interactions through intermolecular hydrogen bonding. This transparent and stable hydrogel exhibited excellent self‐healing properties and injectability, enabling smooth tissue injection using a needle. Subsequently, the biocompatibility of ODE–ME hydrogels is addressed. in vivo experiments confirmed the diverse physiological activities of the hydrogel, including its remarkable antibacterial, antioxidant, and anti‐inflammatory properties. Furthermore, the efficacy of peritoneal cavity injections in preventing postoperative peritoneal adhesions in mice with MetS is investigated. The ODE–ME hydrogel significantly prevented abdominal adhesions and promoted wound healing in mice with MetS. Stable postoperative blood glucose levels were maintained while isolating injured tissues during the healing process. The stable tissue adhesion, biocompatibility, and substantial glucose‐lowering effects of the hydrogel suggest that it is a viable option for preventing postoperative peritoneal adhesions in patients with MetS.

## 2 Results and Discussion

### 2.1 Preparation of ODE–ME Hydrogels

**Figure 1:**
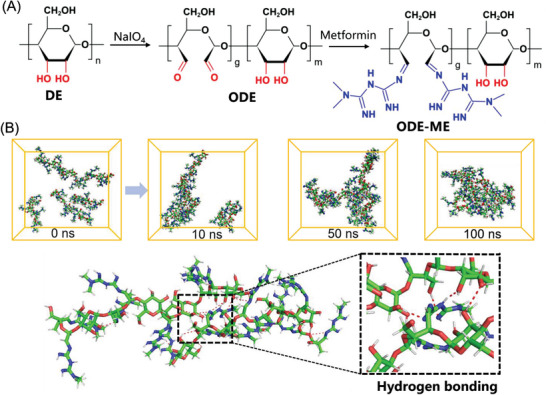
A) Synthesis of ODE–ME hydrogels. B) MD simulation in a water environment within 50 ns, and hydrogen bonding between ODE–ME molecules.

**Figure** **1**A illustrates the synthesis of ODE–ME hydrogels. In the first step, DE is oxidized by sodium periodate under an atmosphere of nitrogen, resulting in ODE with multiple active aldehyde groups. In the second step, small‐molecule ME is grafted onto the molecular chain of ODE through a Schiff base reaction involving the aldehyde group of the ODE molecular chain and the primary amine of ME. The resulting ODE–ME is subjected to thorough dialysis and lyophilization. Notably, upon re‐dissolving the ODE–ME powder in water, the end product is transformed into a transparent, self‐supporting hydrogel (Figure S1, Supporting Information). In comparison, the hydrogel showed partial turbidity at 20 wt.%, possibly owing to the saturation of ODE–ME dissolution. ODE–ME synthesis was further analyzed using gel permeation chromatography (GPC), revealing an Mw of 21133 Da (Figure S2, Supporting Information).

### 2.2 Molecular Dynamics

To understand the potential gelation process of the ODE–ME hydrogel better, AAMD simulations were employed for microscopic characterization. Initially, an ODE–ME molecular fragment with a degree of polymerization of 5 was constructed for the calculations (Figure S3, Supporting Information). The primary focus was on studying the dynamic processes and interactions among the molecules. Five molecules were randomly placed in a 50 Å cube filled with an aqueous solution, which was equilibrated at 298.15 K for 1 ns. Subsequently, a 100 ns molecular dynamics (MD) simulation was conducted, resulting in the formation of the final structure. The MD simulation results were collected at intervals of 0, 10, 50, and 100 ns, with the water molecules hidden for clarity. At 50 ns, all molecules were clearly entangled. These findings indicate that the ODE–ME molecules readily formed cross‐links with each other (Figure 1B). Furthermore, structural insights derived from the final simulations suggest that the formation of cross‐links between the ODE–ME molecules may be attributed to the hydrogen bonding between the guanidine and hydroxyl groups.

### 2.3 Formation Mechanism

**Figure 2:**
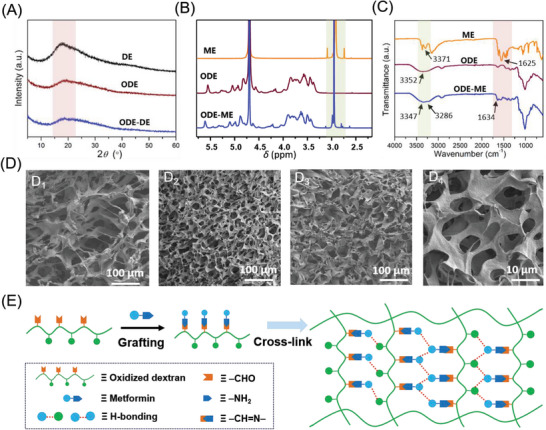
A) XRD of the DE, ODE, and ODE–ME samples. B) 1H NMR and C) FTIR spectra of the ME, ODE, and ODE–ME samples. D) SEM images of (D1) ODE, (D2) ODE–ME (10 wt.%), and (D3 and D4) ODE–ME (15 wt.%). E) ODE–ME hydrogel formation.

To investigate the properties of the ODE–ME hydrogels further, multiple characterization methods were employed to study their molecular structures. The X‐ray diffraction (XRD) analysis of pure DE reveals a dispersion peak within the 2*θ* range of 10°–60°, indicative of its amorphous structure, along with a distinct absorption peak at 2*θ* = 17.5°. The XRD patterns of ODE and ODE–ME resemble that of DE, albeit a significantly reduced absorption peak at 2*θ* = 17.5° (**Figure** **2**A). These findings suggest that both ODE and ODE–ME are amorphous solids with structurally similar underpinnings. Compared to the original characteristic peak of DE (δ 3.2–4.0) in the hydrogen nuclear magnetic resonance spectrum (^1^H NMR), ODE exhibits numerous new proton peaks near δ 5.0 (Figures S4, Supporting Information: Figure 2B). Furthermore, the oxidation degree measured by hydroxylamine hydrochloride potentiometric titration method of ODE was 36.2 ± 2.5%. This indicates the successful disruption of the C‐C bond in this potassium periodate structure during oxidation. The ^1^H NMR spectrum of ODE–ME also reveals the characteristic signal peak of ME (signal 2 = 3.0 ppm; ‐N(CH_3_)_2_), providing further confirmation of the formation of the ODE–ME compound.

Notably, the FTIR spectrum of ODE–ME exhibits an absorption peak at 1634 cm^−1^, corresponding to the characteristic imine stretching vibration (‐C = N‐) (Figure 2C), confirming the formation of dynamic Schiff bonds. For ME, the double absorption peaks near 3371 cm^−1^ and 3296 cm^−1^ represent symmetric and antisymmetric stretching vibration peaks of ‐NH_2_. The stretching vibration absorption peak of C = N appears at 1625 cm^−1^. Notably, in the absence of hydrogen bonding, the ‐OH peak in ODE would typically appear around 3352 cm^−1^. However, in ODE–ME, the ‐OH peaks broadened and shifted to lower wavenumbers. The original C‐O stretching peak in DE (Figure S5, Supporting Information) shifted to lower wavenumbers in both ODE and ODE–ME, measuring 1012 cm^−1^ and 1005 cm^−1^, respectively. Additionally, at higher temperatures, the tendency to break the hydrogen bonds between the groups weakens.^[^ ^10^ ^]^ In the variable‐temperature FTIR spectrum, the position of the OH peak in ODE–ME shifted to higher wavelengths with increasing temperature, whereas that of C = N remained nearly constant (Figure S6, Supporting Information). These observations collectively suggest the existence of stronger hydrogen‐bonding interactions between the ODE–ME molecules.

Considering the results of the AAMD simulations, the physical cross‐linking facilitated by hydrogen bonding likely contributed to the formation of the characteristic three‐dimensional structure observed in hydrogels. Typically, hydrogels possess a common three‐dimensional framework with significant water content. After freeze drying, the pore structure within the framework becomes visible during SEM. As shown in Figure 2D_1_, the original freeze‐dried ODE sample exhibited irregular pore walls and loose pores. Conversely, SEM images of ODE–ME samples reveal numerous microstructures with regular shapes, including continuous pore walls and micropores with sizes ≈5 µm (Figure 2D_2_‐D_4_). These observations suggest that the molecular chains of ODE grafted with ME are more prone to cross‐linking, leading to the establishment of a three‐dimensional network and gelation of the solution. These results provide additional evidence to support the formation of ODE–ME hydrogels (Figure 2E).

### 2.4 Tissue‐Adhesive Performance of ODE–ME Hydrogels

**Figure 3:**
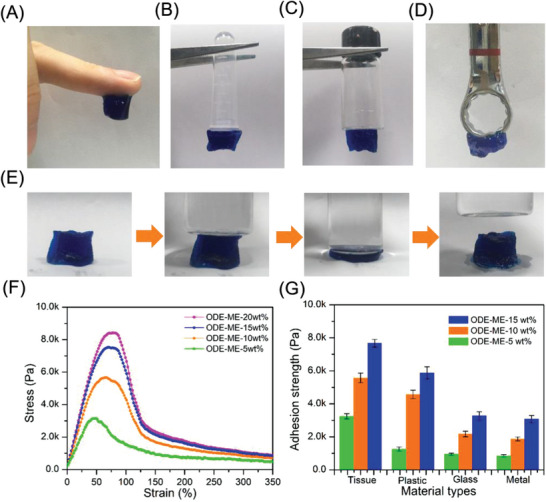
ODE–ME hydrogels can adhere to various substrates: A) human skin B) plastic, C) glass, and D) metal. E) Compression tests on hydrogels. The material was stained using methylene blue. E) In vitro adhesion tests on hydrogels. F) Adhesion strength of hydrogels with different materials.

The molecular chains of the ODE–ME hydrogels are rich in amino and hydroxyl groups, providing these hydrogels with distinctive adhesive properties. The prepared hydrogel demonstrated the ability to adhere to human skin without causing any irritation or inflammation of the skin tissue, making it suitable for potential applications in dressings (**Figure** **3**A). Furthermore, as depicted in Figure 3B–D, the ODE–ME hydrogels exhibited exceptional adhesion to various substrates, such as plastic, glass, and metal. They maintained adhesion for longer durations (2 h) without detachment. Additionally, these hydrogels recovered their original shape after being subjected to external compression without undergoing significant deformation (Figure 3E). Compression test results show that the compressive strength of the ODE–ME‐15wt.% hydrogel reached 20 KPa under a strain of 90%. Although this strength may be lower than that of other tough hydrogels, it demonstrates the capability of the hydrogel to self‐recover at a strain of 80%.^[^ ^11^ ^]^ Figure S7 (Supporting Information) shows the elasticity of the hydrogel, with limited hysteresis after the first cycle. The second and third cycles almost overlapped with no substantial plastic deformation or loss of strength. These findings indicate that this stable adhesive material has broad application potential.

To gain deeper insight into hydrogel adhesion, we conducted adhesion tests using animal tissues, instead of skin, as substrates. As shown in Figure S8 (Supporting Information), regardless of whether the hydrogel was tilted or inverted, it firmly adhered to the peritoneal surface of the abdominal wall or cecum and effectively bonded the two peritoneal surfaces. An interaction between the aldehyde groups in ODE and the amine groups in the damaged tissue is generally observed, and ODE–containing hydrogels can extend to the irregular surface of the damaged tissue and promote epithelial cell adhesion.^[^ ^12^ ^]^ Subsequently, a stable tensile force was applied to the sample to which the tissues were bonded. When the strain reached a specific value, the two tissues began to separate and detach gradually. The results revealed that the adhesion strength reached 3.2 KPa for ODE–ME‐5wt.% and 5.7 KPa for ODE–ME‐10wt.%, exhibiting a 1.7‐fold difference between them. As shown in Figure 3E, the adhesive strength was 7.8 KPa for ODE–ME‐15wt.% and 8.2 KPa for ODE–ME‐20wt.%. These results suggest that the bond strength is closely related to the hydrogel concentration and gradually saturates after reaching 15 wt.%. Furthermore, ODE–ME‐15wt.% hydrogels exhibited strong adhesion to tissues and plastics, whereas all ODE–ME hydrogels displayed significantly lower adhesion to glass and metals (Figure 3F). This may be attributed to the smoother surfaces of the latter two materials, to which the gel adhered less readily.

### 2.5 Rheological Properties of ODE–ME Hydrogels

**Figure 4:**
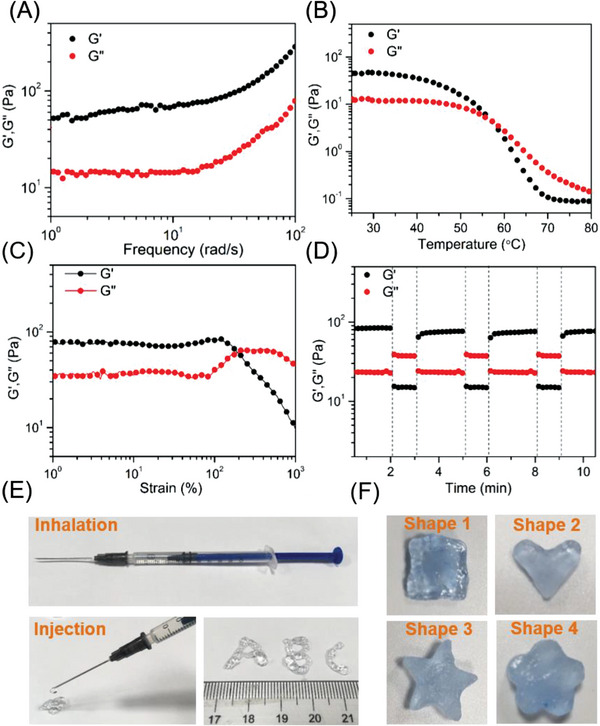
Variation in the storage (G') and loss moduli (G") of the ODE–ME hydrogel in A) frequency sweep, B) temperature sweep, C) strain sweep, and D) continuous step strain tests. E) Injection behavior and F) plasticity of the ODE–ME hydrogel. The material was stained using methylene blue.

We conducted typical rheological tests to further explore the characteristics of the hydrogels with the ME hydrogel concentration fixed at 15 wt.%. As depicted in **Figure** **4**A, the storage modulus (G') of the hydrogel consistently exceeded the loss modulus (G') across the entire frequency scan range, and both values increased steadily with increasing frequency. At the frequencies of 1 and 10 rad s^−1^, the G values of the hydrogels reached 200 and 350 Pa, respectively. Compared to other robust hydrogels based on hydrogen bonding, the prepared ODE–ME‐15wt.% hydrogel exhibited a softer profile. Moreover, the values of G' and G“ remained relatively stable within the temperature range of 25–55 °C (Figure 4B). Beyond 55 °C, G' decreased as temperature increased, eventually falling below G”, indicating the gradual breakdown of hydrogen bonding. Further heating led to the loss of the self‐supporting state of the ODE–ME‐15wt.% hydrogel, resulting in a gel–sol transition and its transformation into a liquid state (Figure S9, Supporting Information). This hydrogel maintained a normal gel state under standard conditions (37 °C; low frequency).

Furthermore, in the shear strain test, the G′ values of all hydrogels decreased significantly as the stress increased, indicating that higher strain levels disrupt the reversible non‐covalent bonds within the material (Figure 4C). Throughout this process, G′ values consistently exceeded G″ values, and the gel‐sol transition point of the ODE–ME‐15wt.% hydrogels was 120%. The reversible hydrogen bonding makes the hydrogel conducive for self‐healing. In step‐strain measurements of the ODE–ME‐15wt.% hydrogels, the G′ value surpassed G″ at a small strain of 0.1% (frequency = 1.0 Hz). As the strain reached 500%, the G' value rapidly declined, falling well below G,’ indicating a gel‐sol transition. On returning to a 0.1% strain, the G’ value quickly exceeded G″ within 10 s, indicating gel regeneration. This entire process could be repeated for at least three cycles (Figure 4D), and G' and G" values at low strains (0.1%) consistently exhibited rapid recovery. Notably, reversible hydrogen bonding rendered the hydrogel injectable and moldable. Tests confirmed that a standard syringe (volume: 1 mL) could be drawn in the ODE–ME‐15wt.% hydrogel and smoothly injected into any site via a needle hole (Figure 4E). Additionally, the hydrogel was molded into specific shapes and maintained for nearly 24 h without any external intervention (Figure 4F). These remarkable properties make this hydrogel suitable for a wide range of applications, especially in scenarios involving precise injections such as in vivo injuries.

### 2.6 Cytocompatibility and Hemocompatibility of ODE–ME Hydrogels

Owing to the presence of hydrophilic groups such as guanidinium and hydroxyl groups that exhibit a strong affinity for water molecules in ODE–ME‐15wt.%, the hydrogel demonstrated water‐absorption properties. As shown in Figure S10 (Supporting Information), ODE–ME‐15wt.% rapidly achieved a dissolution rate of 610% within the first hour, reaching 700% after 3 h of swelling absorption. After 4 h, the hydrogel's water‐absorption capacity gradually saturated, and the swelling rate stabilized near 760%. Furthermore, ME was bound to ODE via an imine bond, a dynamic and reversible chemical bond, enabling its release under specific conditions. The release profile of the hydrogel indicates that ME was continuously released throughout the experiment at physiological pH (pH = 7.4), with cumulative release reaching equilibrium (51.8% ± 3.0%) at 90 h.^[^ ^13^ ^]^ These results indicate that the ODE–ME hydrogels are capable of fulfilling the requirements of rapid blood absorption and drug release following injury.

**Figure 5:**
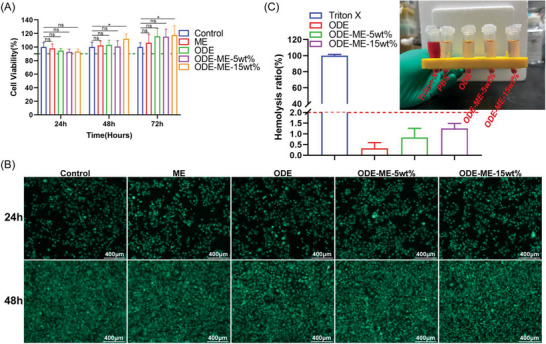
Biocompatibility of ODE–ME hydrogels. A) CCK8 assays evaluating the in vitro cytotoxicity of ODE–ME hydrogels (*n* = 6). B) Representative pictures of calcein/PI live/dead staining of Met‐5A cells (live cells are stained green). C) Representative pictures and quantitative statistical data of the hemolysis rate of ODE–ME hydrogels (*n* = 6). Scale bar: 400 µm. Here, ns indicates no statistical difference, *p < 0.05.

Biocompatibility, including cytocompatibility and haemocompatibility, is a critical factor in biomaterial applications.^[^ ^14^ ^]^ In vitro cytotoxicity was assessed using Met‐5A cells, and hemolytic activity was evaluated using rat erythrocytes. The CCK8 assay results revealed that the cell viability in each group was >90%, and neither ODE nor ME was significantly cytotoxic compared to the control group (**Figure** **5**A). The ODE–ME hydrogels did not exhibit any notable inhibitory effects on the proliferation of Met‐5A cells. Over time, the hydrogels mildly promoted the proliferation of human mesothelial cells. Calcein–propidium iodide (PI) staining was performed to further assess the cytocompatibility of the hydrogels. Using calcein‐acetoxymethyl (AM), Living cells were stained green with calcein acetoxymethyl. Figure 5B shows that the morphology of the Met‐5A cells in each group remained normal. Consistent with the previous CCK8 assay results, cell proliferation in each group was not significantly disrupted after 24 and 48 h. Peritoneal mesothelial cell proliferation plays a pivotal role in the repair of peritoneal injuries, and inhibition of their proliferation may hinder the healing process.^[^ ^15^ ^]^ The results of the in vitro hemolysis experiments are shown in Figure 5C. No evident hemolysis was observed after co‐incubation the hydrogels with rat erythrocyte suspensions, with hemolysis rates in each group remaining below 2%. Following the injection of ODE–ME‐15wt.% hydrogels into the abdominal cavity of the rats, blood samples were collected for routine examinations. As depicted in Figure S11 (Supporting Information), at 7 and 14 d after injection of ODE–ME hydrogels, the white blood cell count, platelet count, red blood cell count, and hemoglobin levels in rats remained within the normal range, with no occurrence of hemolysis. In summary, the ODE–ME hydrogels demonstrate biocompatibility and are suitable for subsequent in vivo experiments.

### 2.7 in vivo Degradation and Organ Toxicity of the ODE–ME Hydrogels

Understanding whether hydrogel degradation occurs in vivo can not only help evaluate its biocompatibility but also estimate its retention time in the abdominal cavity. The suitability of an antiadhesive hydrogel material is determined based on the following characteristics: maintenance of its form in vivo for a certain period and degradability.^[^ ^7^, ^14^ ^]^ The formation of abdominal adhesions begins 3–5 days postoperatively, with 5–7 days being a peak adhesion period.^[^ ^14b^ ^]^ Therefore, the hydrogel should remain in the abdominal cavity for more than 7 days. The FITC‐labeled ODE–ME‐15wt.% hydrogel was intraperitoneally injected into SD rats with a cecum abrasion‐sidewall defect. in vivo imaging revealed that the fluorescence signals became weaker and the red fluorescence area gradually decreased with time, suggesting that the hydrogel was continuously degrading (Figure S12, Supporting Information). At 7 and 10 days postoperatively, the residual rates of the intraperitoneal hydrogel were 22.59% and 2.97%, respectively. After 14 d, the hydrogel was degraded.

The effects of the hydrogel degradation products on the liver, kidneys, lungs, and other organs cannot be ignored. Therefore, hematoxylin and eosin (H&E) staining was performed to elucidate the adverse effects of the ODE–ME hydrogels on the heart, liver, spleen, lungs, and kidneys. Figure S13 (Supporting Information) shows that no organ toxicity was observed in any of the groups. Furthermore, the myocardial structure was unaffected with no evident inflammatory cell infiltration. In addition, the structures of the hepatic sinusoids and lobules, the distribution of the white and red pulps in the spleen, and the alveolar and bronchial structures were normal. Moreover, there was no evidence of necrosis, edema, or inflammatory cell infiltration. Finally, the renal tubular arrangement and glomeruli were normal. Blood biochemical tests revealed that intraperitoneal injection of the ODE–ME hydrogel did not lead to functional damage to the liver and kidneys (Figure S11, Supporting Information). Taken together, the in vivo retention time of the ODE–ME hydrogels was >7 days, covering the key period of peritoneal adhesion development. Furthermore, they degraded within 14 days, and the degradation products exhibited no evident organ toxicity in vivo.

### 2.8 Antibacterial Properties of the ODE–ME Hydrogels

**Figure 6:**
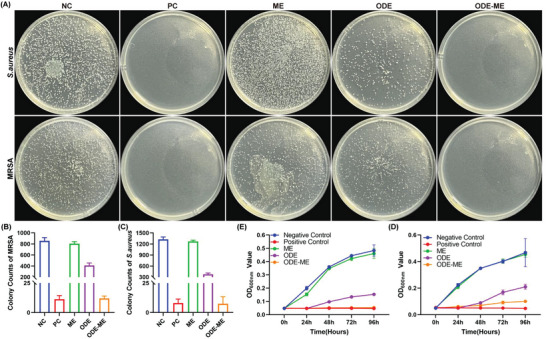
Antibacterial properties of the ODE–ME hydrogels. A) Representative images and B,C) quantitative statistical data of the antimicrobial activity of the ODE–ME hydrogel against methicillin‐resistant *Staphylococcus aureus* (MRSA) and *Staphylococcus aureus* (*n* = 3). Time‐dependent antibacterial test for D) MRSA and E) *S. aureus* (*n* = 3). NC: negative control; PC: positive control.

The presence of gut microbes poses a risk of infection after abdominal surgery involving the gastrointestinal tract. Peritoneal injury complicated by a bacterial infection can be severe. Bacterial contamination was associated with adhesion formation.^[^ ^16^ ^]^ Patients with MetS have long‐term glucose metabolism disorders and chronic inflammation, which may impair immune cell function. Furthermore, these patients may experience surgical trauma and stress during surgery. Moreover, infections caused by difficult‐to‐treat antibiotic‐resistant bacteria, such as MRSA, are becoming more frequent. Subsequently, patients, particularly obese patients, are more prone to postoperative infections, particularly those with obesity.^[^ ^3^, ^17^ ^]^ Previous studies have reported that peritoneal bacterial contamination can result in macrophage and B‐cell assembly in the injured area, which acts on the EGF receptor by binding to its ligand in mesothelial cells. This, in turn, activates myofibroblasts in the mesothelial cells, eventually resulting in adhesion formation.^[^ ^18^ ^]^ A surface antibacterial assay was performed to determine the antibacterial activity of the ODE–ME hydrogels against MRSA, *Staphylococcus aureus*, and *Escherichia coli*. When the ODE–ME hydrogel was co‐cultured with MRSA and *S. aureus*, there was a significant decrease in the number of bacterial colonies on Luria–Bertani (LB) agar (**Figure** **6**A–C). Bacterial density was determined by measuring the absorbance of the LB broth at 600 nm. Figure 6D,E illustrate that ODE exhibited some degree of antibacterial effect on both MRSA and *S. aureus*; however, ME did not effectively inhibit the growth and reproduction of these bacteria. Notably, after co‐culturing MRSA and *S. aureus* with the ODE–ME hydrogels, their densities decreased significantly. Within 96 h, the ODE–ME hydrogel exhibited good antibacterial ability, and over time, the antibacterial effect of the ODE–ME hydrogel was better than that of the ODE hydrogel, suggesting that the antibacterial effect of the hydrogel formed by the combination of ODE and ME was enhanced. Similarly, some degree of antibacterial effect was observed in the antimicrobial experiment with *E. coli* (Figure S14, Supporting Information).

ODE contains aldehyde groups in its molecular chain; therefore, it exhibits some degree of antibacterial activity,^[^ ^12^, ^19^ ^]^ which is consistent with our study results. When ODE was combined with ME to form a hydrogel, the antibacterial activity of the hydrogel was significantly enhanced, possibly because ME could affect the membrane potential of bacteria and lead to ion channel dysfunction in the cell membrane. Furthermore, ME not only improved colonic barrier dysfunction in aged rats with sepsis but also enhanced the antibacterial effects of drugs.^[^ ^20^ ^]^ Therefore, the combination of ME and ODE to form an ODE–ME hydrogel may exert an enhanced antibacterial effect, thereby significantly improving the antibacterial activity of the materials prepared in this study.

### 2.9 Anti‐Fibroblast Adhesive and Antioxidant Properties of the ODE–ME Hydrogels

**Figure 7:**
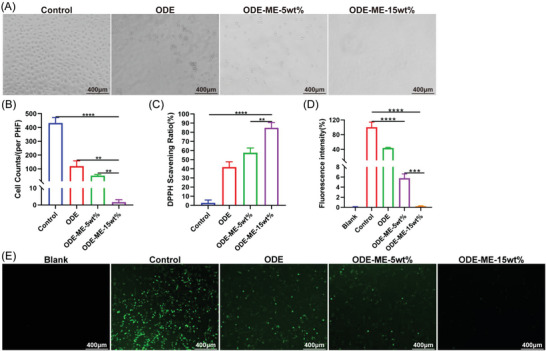
Anti‐fibroblast adhesive and antioxidant properties of the ODE–ME hydrogels. A) Representative images and B) quantitative statistical data of the anti‐L929 fibroblast adhesive property of the ODE–ME hydrogels (*n* = 3). C) Quantitative analysis of the 2,2‐diphenylpicrylhydrazyl radical scavenging rate of the ODE–ME hydrogels (*n* = 3). D) Quantitative statistical analysis data and E) representative images of the ROS scavenging ability of the ODE–ME hydrogels in RAW264.7 cells (Emission wavelength: 525 nm; Excitation wavelength: 488 nm; *n* = 3). Scale bar: 400 µm. **p < 0.01, ***p < 0.001, ****p < 0.0001.

The mesothelial–mesenchymal transition of peritoneal mesothelial cells is a crucial mechanism underlying adhesion formation.^[^ ^21^, ^22^ ^]^ When peritoneal mesothelial cells differentiate into fibroblasts, the collagen and inflammatory marker levels increase, promoting the development of fibrous adhesions. Inhibition of fibroblast formation may help decrease adhesion formation. Fibroblasts were cultured in culture plates covered with ODE–ME hydrogels. Compared to the control group, the number of adhered fibroblasts was significantly lower in the hydrogel group (**Figure** **7**A,B). Furthermore, as the concentration increased, the effect of the hydrogel became stronger with fewer adhered fibroblasts.

In the early stages of peritoneal injury, neutrophils and macrophages are recruited to the injury site and reactive oxygen species (ROS) accumulate.^[^ ^23^ ^]^ Patients with MetS exhibit increased ROS production owing to disturbances in glucose and lipid metabolism, chronic inflammation, mitochondrial dysfunction, increased inflammatory factor levels, and surgical stimulation. When there is an imbalance in the antioxidant mechanism, excessive ROS not only impairs wound healing, but also damages mesothelial cells, possibly increasing adhesion.^[^ ^4^, ^24^ ^]^ Therefore, the inhibition of excessive oxidative stress may help decrease adhesion and promote healing of the injured peritoneum. The 2,2‐diphenylpicrylhydrazyl scavenging rates of the ODE–ME‐5wt.% and ODE–ME‐15wt.% hydrogels were 57.6% and 84.9%, respectively (Figure 7C). Furthermore, the scavenging rates of the ODE–ME‐5wt.% and ODE–ME‐15wt.% hydrogels were 94.2% and 99.8%, respectively (Figure 7D,E). Consistent with previous findings, the higher the hydrogel concentration, the more pronounced was the antioxidant effect. In summary, ODE–ME hydrogels exhibited anti‐fibroblast adhesive and antioxidant effects and played a favorable role in preventing complex MetS‐based postoperative peritoneal adhesions.

### 2.10 Effects of the ODE–ME Hydrogels in Preventing MetS‐Complicated Peritoneal Adhesions

**Figure 8:**
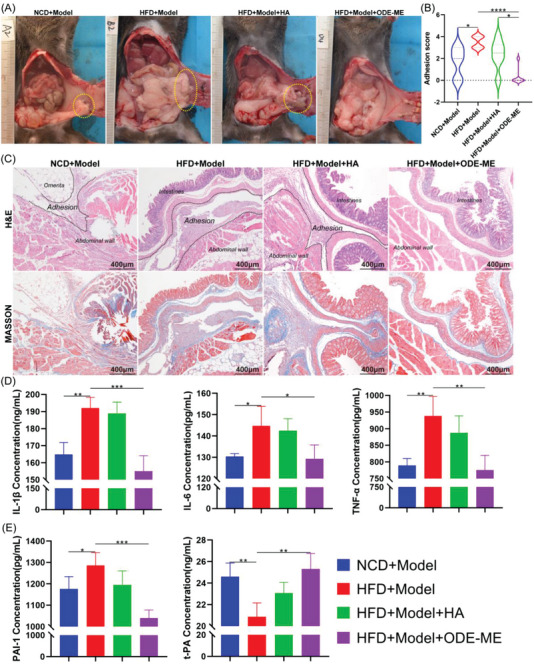
Performance of the ODE–ME hydrogel in preventing peritoneal adhesions in MetS. A) Representative images of ischemic button adhesions in the abdominal cavity on postoperative day 7 and B) adhesion scores of each group (*n* = 6). C) Representative H&E and Masson staining images of each group on postoperative day 7. D,E) Serum IL‐1β, IL‐6, TNF‐α, PAI‐1, and t‐PA levels on postoperative day 7 (*n* = 4). Scale bar: 400 µm. *p < 0.05, **p < 0.01, ***p < 0.001, ****p < 0.0001.

C57BL/6J mice were fed a high‐fat diet (HFD) to induce the metabolic syndrome. An intraperitoneal ischemic button adhesion model was developed in mice to evaluate whether the adhesions in mice with MetS were more severe than those in normal mice and whether the ODE–ME hydrogel could prevent complex MetS‐induced postoperative peritoneal adhesions. Compared with normal control diet (NCD)‐fed mice, HFD‐fed mice not only gained weight, but also became glucose‐intolerant and insulin‐resistant, suggesting the successful development of the MetS model (Figures S15 and S16, Supporting Information). The average adhesion scores of the HFD + Model and NCD + Model groups were 3.5 (*n* = 6) and 1.7, respectively. Furthermore, severe adhesions were observed in the abdominal cavity involving the ischemic buttons of the abdominal wall, omentum, intestinal wall, mesentery, and bladder (**Figure** **8**A,B). Studies have reported that the incidence of adhesions is higher in obese patients than in non‐obese patients.^[^ ^4^ ^]^ In the present study, we observed that the incidence and severity of postoperative peritoneal adhesions were higher in MetS mice than in control mice. Although treatment with the HA hydrogel slightly relieved peritoneal adhesions in mice with MetS (average score of 2.3), the effect of the HA hydrogel was unstable, with severe adhesions still observed in some mice (Figure 8A,B). Because of the interaction between the aldehyde groups in ODE and the amine groups in wound tissues, hydrogels can extend to the irregular surfaces of peritoneal injuries and promote epithelial cell adhesion.^[^ ^12^ ^]^ Treatment with the ODE–ME hydrogel significantly decreased abdominal adhesions, with an average score of 0.3.

H&E and Masson staining (Figure 8C) revealed that the adhesions between the omentum and abdominal wall were thinner and the collagen fiber content was lower in the NCD + Model group. In contrast, the adhesions in the HFD + Model group were thicker and had higher collagen fiber content. Treatment with the HA hydrogel slightly decreased abdominal wall thickness and collagen fiber content. However, after treatment with the ODE–ME hydrogel, adhesions were significantly decreased, visceral and parietal peritoneal structures were intact, and collagen fiber deposition was decreased.

In general, compared with patients without MetS, those with MetS present with chronic inflammation and higher levels of markers, including interleukin (IL)−6, tumor necrosis factor (TNF)‐α, and IL‐1β. MetS is characterized by oxidative stress, blood hypercoagulation, and fibrinolytic imbalance.^[^ ^6^ ^]^ Stimulation from surgical injury will further aggravate inflammatory responses and fibrinolytic imbalance, leading to the development of more severe adhesions.^[^ ^24^, ^25^ ^]^ An enzyme‐linked immunosorbent assay was performed to determine whether the ODE–ME hydrogel could alleviate chronic inflammation and fibrinolytic imbalance in mice with MetS after surgery. Compared with the NCD + Model group, serum IL‐6, IL‐1β, TNF‐α, and plasminogen activator inhibitor‐1 (PAI‐1) levels were higher in the HFD + Model group; however, tissue‐type plasminogen activator (t‐PA) levels were lower; this suggests that mice with MetS have more severe postoperative inflammation and lower fibrinolytic activity, which are closely associated with adhesion severity (Figure 8D,E). After treatment with the ODE–ME hydrogel, serum IL‐6, IL‐1β, TNF‐α, and PAI‐1 levels decreased, whereas t‐PA levels increased. These results suggest that the ODE–ME hydrogel can alleviate excessive postoperative inflammatory responses and increase fibrinolytic activity in mice with MetS, thereby decreasing adhesion formation.

**Figure 9:**
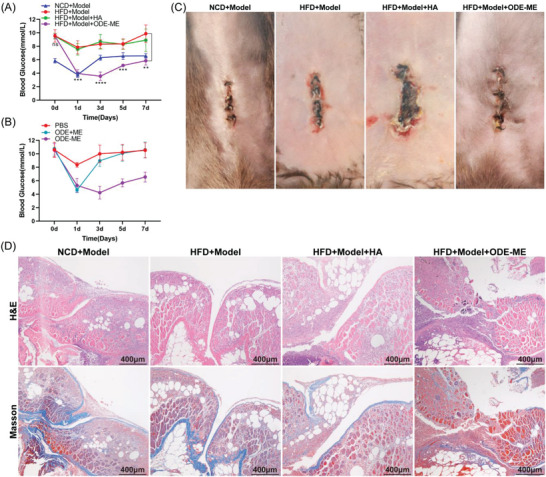
Hypoglycemic and incision healing properties of the ODE–ME hydrogels in mice with MetS. A,B) Postoperative fasting blood glucose levels in mice in each group at different time points. C) Representative images of postoperative abdominal incision healing in the ischemic button model. D) Representative H&E and Masson staining images of the postoperative abdominal incisions in the ischemic button model. Scale bar: 400 µm. **p < 0.01, ***p < 0.001, ****p < 0.0001.

Next, we evaluated the effect of the ODE–ME hydrogels on postoperative blood glucose levels. Compared to the HFD + Model group, the ODE–ME hydrogel group showed decreased fasting blood glucose levels in mice with MetS within a certain period and maintained blood glucose at a relatively stable level (**Figure** **9**A). Equal amounts of ODE hydrogel and ME solution were successively injected into the abdominal cavity of the same mouse with MetS to evaluate the hypoglycemic effect when ODE and ME did not form an ODE–ME hydrogel. These findings are summarized in Figure 9B. Compared with the control group (PBS), when ODE and ME did not form an ODE–ME hydrogel, the ME solution was quickly absorbed into the blood and could only exert some degree of hypoglycemic effect within 2–3 days. On the other hand, when both ODE and ME were combined to form the ODE–ME hydrogel, the strength of the hydrogel allowed it to remain in the body for some period and continuously release ME during the degradation process; therefore, the hydrogel could control hyperglycemia for a longer period.

Compared to patients without MetS, incision healing is more challenging in patients with MetS undergoing surgery owing to insulin resistance and chronic inflammation. As shown in Figure 9C, compared with the NCD + Model group, the abdominal incisions in the HFD + Model group exhibited poor healing capacity with suppuration, swelling, and hyperemia. Treatment with HA hydrogel did not improve incision healing. However, no evident dehiscence or hyperemia was observed in the abdominal incisions after treatment with ODE–ME hydrogel. H&E and Masson's staining (Figure 9D) revealed that the incisions in the mice of the NCD + Model group healed well, with hyperplastic chronic granulomas and collagen fibrous tissue connections. However, the incisions in the mice in the NCD + Model and HFD + Model + HA groups did not heal in the hyperplastic tissue. Furthermore, in the HFD + Model + ODE–ME group, chronic hyperplastic granulomatous tissues were connected to the incision site. The ODE–ME hydrogel promoted healing of surgical incisions in patients with MetS, which may be related to its ability to regulate glucose metabolism and excessive inflammatory responses.

The ODE–ME hydrogel also exhibited excellent antiadhesive effects in a rat cecum abrasion‐sidewall defect model, reducing the deposition of collagen fibers (Figure S17, Supporting Information). Compared to the model group, the ODE–ME hydrogel reduced the expression of the fibrosis marker proteins Collagen I and SMA in adhesion tissues, suggesting that the ODE–ME hydrogel can inhibit fibrosis (Figure S18, Supporting Information). HIF1α and PKM2 expression was upregulated in the adhesion tissues of the model group compared with those of the sham operation group. The upregulated expression of HIF1α and PKM2 was reversed after ODE–ME hydrogel treatment. This finding suggests that the mechanism by which the ODE–ME hydrogel prevents postoperative peritoneal adhesion formation is related to the inhibition of the HIF1α/PKM2 signaling pathway. In summary, the ODE–ME hydrogel effectively prevented complex MetS‐associated peritoneal adhesions, alleviated insulin resistance and inflammation, and improved low‐fibrinolytic activity.

## 3 Conclusion

This study developed a multifunctional ODE–ME hydrogel via the Schiff base binding of ODE and ME to address the issue of severe postoperative adhesions in patients with MetS. The prepared hydrogel exhibited excellent self‐healing, injectable, and tissue‐adhesive properties owing to multiple intramolecular dynamic hydrogen bonds. In particular, the hydrogel exerted an excellent antibacterial effect, with significant inhibition of gram‐negative *E. coli* and gram‐positive *S. aureus*. Furthermore, in postoperative peritoneal adhesion experiments conducted on mice with MetS, the hydrogel exhibited superior performance in preventing MetS‐complicated peritoneal adhesions, mitigating insulin resistance and hyperinflammatory responses, and increasing fibrinolytic activity compared to control groups. Importantly, the ability of a hydrogel to promote postoperative incision healing is an important factor that should be considered when developing a hydrogel as a therapeutic material for patients with MetS undergoing surgery in a clinical setting.

## 4 Experimental Section

### Materials

Dextran (Mw = 400 kDa), sodium periodate, and sodium carbonate were purchased from Sigma‐Aldrich Inc. (Shanghai, China). ME was purchased from Macklin Biochemical Co. Ltd. (Shanghai, China). All other chemicals were purchased from Aladdin Reagent Co., Ltd. (Shanghai, China). All chemicals were used without purification, and deionized water was used for all experiments.

### Preparation of Hydrogels

First, 10 g of Dextran was dispersed in 50 mL of ethanol, and then, 50 mL of distilled water containing 10 g of sodium periodate was added to the solution. The mixture was stirred magnetically at 25 °C in the dark for 6 h. Subsequently, 5 mL ethylene glycol was added to stop the reaction. After stirring for another 2 h, 1.0 L ethanol was poured into the reaction mixture. The prepared oxidized dextran was then dialyzed with deionized water for three days and freeze‐dried.

ODE–ME hydrogels were prepared by in situ gelation of ODE and ME. First, 0.40 g of ME was added to the ODE solution (10 wt.%; 100 g) and allowed to react for 1 h to obtain ODE–ME hydrogels. The hydrogels were freeze‐dried and ground into powder. Finally, different concentrations of ODE–ME powder were completely dissolved in deionized water, stirred for 1 h at room temperature (≈25 °C), and left to stand for 0.1 h. The states of the hydrogels were tested using the vial inversion method.

The degree of oxidation of the ODE samples was determined using hydroxylamine hydrochloride potentiometric titration.^[^ ^26^ ^]^ To this end, first, 0.2 g sample was dissolved in 50 mL of 0.25 m hydroxylamine hydrochloride solution containing 0.01% methyl orange. The mixture was then titrated with 0.1 m NaOH. The aldehyde groups reacted with hydroxylamine hydrochloride to produce hydrochloric acid, which was neutralized using NaOH. The oxidation degree (ODD) was calculated as ODD (%) = (*Δ*V × 0.001 × C_NaOH_×0.5×160)/M_0_ × 100. Here, *Δ*V is the consumed volume of NaOH solution (mL), C_NaOH_ is the concentration of NaOH (mol L^‐1^), M_0_ is the weight of ODE, and the number 160 represents the molecular weight of repeating units (g mol^−1^).

### All‐Atom Molecular Dynamics (AAMD)

The ODE–ME polymer fragment was built using Gaussian View 16 and geometrically optimized using the B3LYP method and the 6–31G (d, p) basis set. Subsequently, electrostatic potential (ESP) was calculated using Guassian View 16 by employing the HF/6‐31G* method and basis set. All‐atom molecular dynamics (AAMD) simulations were performed using GROMACS (version 2020.6) software tool and generalized amber force field (GAFF) and TIP3P water models.^[^ ^27^ ^]^ Initially, five molecules were packed randomly in a cube with a side length of 50 Å. The system was then minimized using a conjugate gradient algorithm. The temperature and volume of the entire system were equilibrated by running at a constant pressure (NPT) for 1 ns. The final run in the NPT continued for 100 ns. This process maintained a temperature of 300 K and a pressure of 1 bar for the isothermal–isobaric (constant NPT) combination. At the end of the simulation, the binding structures of these molecules at different times were extracted and displayed using VMD software package.

### Characterization

The number‐average molecular weight (Mn) and weight‐average molecular weight (Mw) of the ODE–ME hydrogels were determined via gel permeation chromatography (GPC) using a UK PL‐GPC 220 instrument and US Wyatt Model: DAWN HELEOS II.^[^ ^28^ ^]^

The dextran, oxidized dextran, ME, and ODE–ME samples were vacuum dried for 48 h and mixed with infrared light‐baked KBr crystals. They were then dried again and ground to powder. At 25 °C, FTIR spectra of all samples were collected using an iS50 spectrometer (Thermo Nicolet, USA) in the range of 400–4000 cm^−1^. In particular, the variable temperature FTIR spectra of the ODE–ME samples were analyzed at 25 °C, 40 °C, 55 °C, 75 °C, and 85 °C, respectively.

Hydrogels were obtained by dissolving 1 g of ODE–ME in 9 g of deionized water. Subsequently, the hydrogels were freeze‐dried for 48 h, and their microstructures were observed using scanning electron microscopy (SEM; Quanta 250 FEG, FEI Inc., Czech Republic).

The dextran, oxidized dextran, ME, and lyophilized ODE–ME samples were dissolved in pure D_2_O. The ^1^H NMR spectra of all the samples were recorded on a Bruker AVANCE III spectrometer operating at 400 MHz. Meanwhile, all temperatures were fixed at 25 °C. X‐ray diffraction (XRD) patterns of the samples were collected using an X‐ray diffractometer (D8 Advance, Bruker AXS Co. Ltd., Germany) with Cu‐Kα radiation in the 2*θ* range of 5°–60°.

### Rheological Mechanics

The rheological behavior of the samples was analyzed using a modular compact rheometer (Anton Paar, MCR 302). Notably, the ODE–ME‐15 wt.% hydrogel was used for all tests. The frequency (ω) sweep test is carried out in the range of 0.1–100 rad s^−1^, and the strain (γ) in all the above tests is fixed at 0.5%. Temperature sweep test was carried out in the range of 25–80 °C, and ω was fixed at 0.5 rad s^−1^. Strain test was performed at 1 Hz frequency, and the strain range was 0.1%–1000%. Step strain sweep tests were performed at a constant frequency (1 Hz) with strains of 0.1% and 500%. The test comprised two steps. First, the hydrogel was measured at a low strain of 0.1% for 2 min. Second, the strain was increased to 100% and was maintained for 1 min to damage the hydrogels completely. Furthermore, the high strain was reduced to 0.1% and was maintained for 2 min to observe whether the gel state was restored. The entire test was repeated three times at 25 °C.

### Tissue‐Adhesive Performance Test

ODE–ME‐15wt.% hydrogel was injected into the intestinal and abdominal walls of SD rats, and the adhesion performance of the hydrogel on the peritoneal surface of the intestinal and abdominal walls was observed. The adhesion properties of the hydrogel were studied using a micro‐force biomechanical tester (MTS Insight 30; MTS Company). Briefly, the abdominal wall was cut into rectangles of 10 × 40 mm. Then, 300 µL ODE–ME hydrogels were used to stick the peritoneal surfaces of the two abdominal walls together. The adhesive area was calculated to be 10 × 20 mm. After keeping at room temperature for 15 min, all samples were subjected to a series of lap shear tests using the MTS machine, and the stress–strain data were recorded. The machine was equipped with a load cell of 1000 N operating at a speed of 2 mm min^−1^.

Cylindrical samples with a diameter of 10 mm and length of 10 mm were used for conventional compression and loading–unloading compression tests. The compression rate for both tests was set to 10 mm min^−1^, and the loading–unloading process was repeated after 1.2 min. All the tests were conducted at room temperature.

The engineering stress was calculated as σ = F/S, where S is the sectional area of the specimen and F is the load. The engineering strain was calculated as ε = (l − l_0_)/l_0_ × 100%, where the change in length (l) is related to the initial gauge length (l_0_) of the specimen.

### In Vitro Sustained Release Behavior

ODE–ME‐15 wt.% hydrogel was prepared as a disc‐shaped sample with a diameter of 10 mm and thickness of 5 mm. The samples were then freeze‐dried to obtain a constant weight. These dried gels were immersed in a phosphate‐buffered solution (PBS) with a pH of 7.4 and a concentration of 0.01 mol L^−1^, maintaining a constant temperature of 25 °C.^[^ ^29^ ^]^ At specific time points, the water‐absorbed samples were removed and weighed. The water‐absorbing swelling rate of the ODE–ME hydrogel was calculated as S % = (W_t_ − W_0_)/ W_0_ × 100%, where W_0_ represents the mass of the dry hydrogel and W_t_ indicates the mass of the hydrogel following water absorption. Five parallel experimental groups were used for calculations.

For understanding the in vitro release of ME from ODE–ME‐15wt.% hydrogels, first, 1.0 g of hydrogel (ME: 5.8 mg) was placed in 5 mL of PBS (pH = 7.4) and mixed in a shaker at 37 °C (100 rpm). Then, 500 µL of the upper sustained release solution was withdrawn at regular intervals, and 500 µL of fresh PBS medium was added. The amount of ME released was measured at 233 nm using a UV spectrophotometer (Shimadzu UV‐2450 Spectrometer, Japan). The sustained release of the hydrogel was studied, and the cumulative percentage of ME released was determined at different times. Five parallel experimental groups were used for calculations.

### Cell Lines and Animals

Mouse fibroblast L929, human mesothelial cell line Met‐5A, and a mouse mononuclear macrophage cell line RAW264.7 were purchased from the American Type Culture Collection (ATCC), and the culture conditions were maintained at 37 °C in a 5%‐CO_2_ constant temperature incubator. L929 and RAW264.7 were cultured in Dulbecco's modified Eagle's medium (DMEM) containing 10% fetal bovine serum and 1% penicillin‐streptomycin. Met‐5A was cultured in the M199 medium containing 1.5 g L^−1^ of sodium bicarbonate, 10% of fetal bovine serum, 3.3 nm of epidermal growth factor (EGF), 1% of insulin‐transferrin‐selenium, 0.02 m of HEPES, 400 nm of hydrocortisone, and 1% of penicillin‐streptomycin. SD rats and C57BL/6J mice (both 5–6 weeks old) were purchased from Slack Jingda Laboratory Animal Company (Changsha, China). Animals were housed in an IVC‐grade facility at 20–22 °C and relative humidity of 50–60%. All animal experimental protocols were approved by the Experimental Animal Welfare Ethics Committee of Central South University (CSU‐2022‐0001‐0059 and CSU‐2022‐0001‐0064).

### In Vitro Cytotoxicity Assay

Met‐5A cells were seeded at a density of 5000 cells per well. After 24 h of incubation, each group of ODE–ME hydrogels was added. Cell counting kit‐8 (CCK8) was used at a ratio of 10:1 at 24, 48, and 72 h. After incubation for 1 h, the absorbance at 450 nm was measured using a microwell plate spectrophotometer. Met‐5A cells were plated at a density of 10000 cells per well, and each group of ODE–ME hydrogels to be tested was added to each well. Cells were stained for 30 min using calcein‐AM and propidium iodide (PI) cell viability and cytotoxicity assay kits after culturing for 24 and 48 h. A fluorescence‐inverted microscope was used to observe and capture images.

### In Vitro Hemolysis Test

Blood compatibility test of the ODE–ME hydrogels was performed as previously described.^[^ ^30^ ^]^ Erythrocytes from rat blood were separated via centrifugation at 3000 rpm for 10 min. The obtained erythrocytes were diluted in phosphate‐buffered saline (PBS) to achieve a final concentration of 5%. The hydrogel samples (200 µL) to be tested were added into 1000 µL erythrocyte suspension; 200 µL PBS and 200 µL 0.1% Triton X‐100 were used as the negative and positive controls, respectively. The mixture was incubated at 37 °C for 1 h at a speed of 150 rpm min^−1^ in a rotatory shaker and then centrifuged at 3000 rpm min^−1^ for 10 min; 100 µL of the supernatant was added to a 96‐well plate. The absorbance at 545 nm was measured using a multifunctional microplate reader.

(1) $$\mathrm{Hemolysis}\;\mathrm{ratio}=\left(\mathrm{As}-\mathrm{An}\right)/\left(\mathrm{Ap}-\mathrm{An}\right)\times 100\%$$

where A_s_, A_p,_ and A_n_ absorbances of the ODE–ME hydrogels, positive control group, and negative control group, respectively.

### In Vitro Anti‐Fibroblast Adhesion Test

Anti‐fibroblast adhesion tests were performed as previously described.^[^ ^8b^ ^]^ The L929 cells were plated at a density of 5 × 10^4^ cells per well in a 24‐well plate loaded with 200 µL of ODE–ME hydrogels; 200 µL of PBS solution was used as the control group. The medium was discarded after 18 h of incubation, and the cells were gently rinsed with PBS three times. The plates were observed and photographed using an inverted optical microscope.

### In Vitro Antioxidation and Interactive Oxygen Species Generation Assays

Antioxidant properties of the hydrogels were evaluated by analyzing their scavenging efficiency against α, α‐diphenyl‐β‐picrylhydrazyl (DPPH) free radicals.^[^ ^8b^ ^]^ Briefly, the ODE–ME hydrogels to be tested were added to 100 µm of DPPH methanol solution; PBS solution was used as the control group. After incubation for 1 h at room temperature, the absorbance at 517 nm was measured using an ultraviolet–visible (UV–vis) spectrophotometer. The antioxidative function of the ODE–ME hydrogels was evaluated by detecting the inhibition of reactive oxygen species (ROS) generation in macrophages. Briefly, RAW264.7 cells were plated in a 96‐well plate at a density of 5000 cells per well. The hydrogels to be tested were added and incubated for 24 h. PBS was used as a control. After incubation with 1 mm hydrogen peroxide (H_2_O_2_) for 1 h, the cells were stained using an ROS assay kit. Results were analyzed using images captured by an inverted fluorescent microscope.

### Antibacterial Activity Studies

Methicillin‐resistant *Staphylococcus aureus* (MRSA; ATCC43300), *Escherichia coli* (ATCC25922), and *Staphylococcus aureus* (ATCC25923) were used to test the antibacterial activity of the ODE–ME hydrogels, and vancomycin hydrochloride, ceftazidime, and oxacillin sodium were used as the positive control groups for these three bacteria, respectively. The bacteria were cultured to attain a density of 1 × 10^5^ colony‐forming units (CFUs)/mL in sterile Luria Bertani (LB) liquid medium; 10 µL of the bacterial culture was added to LB medium containing different hydrogel samples. PBS was used as the negative control. After incubation at 37 °C at a speed of 200 rpm min^−1^ in a rotatory shaker, the absorbance at 600 nm of the bacterial solution was measured. After the bacteria were co‐cultured with ODE–ME hydrogels for a certain period, the bacterial solution was spread onto LB agar plates. After incubation at 37 °C for 12 h, the plates were observed and images were captured.

### In Vivo Degradation Test

Fluorescein isothiocyanate (FITC)‐labeled ODE–ME‐15wt.% hydrogel was injected into the abdominal cavity of SD rats with cecum‐side wall abrasion. Hydrogel degradation was evaluated by observing the fluorescence signal intensity in the rats at different time points using an in vivo optical imaging system (Vilber Fusion FX7, France). An automatic blood cell analysis device (XN‐1000‐B1, China) and automatic biochemical analyzer (Catalyst One, USA) were used for routine blood and biochemical examinations of the rats, respectively.

### Prevention of Peritoneal Adhesions Complicated with MetS

A MetS model was constructed as described previously.^[^ ^31^ ^]^ C57BL/6J mice were fed a 60 kcal% high‐fat diet (HFD; D12492, Research Diets) for 16 weeks. Mice in the control group were fed a low‐sugar, low‐fat diet (normal chow diet [NCD], TP23302, and Teluofei). The body weight of the mice was measured regularly, and glucose tolerance and insulin tolerance tests were performed to evaluate model construction. Specifically, 1 g kg^−1^ of glucose and 0.75 U kg^−1^ of insulin were injected intraperitoneally after the mice were fasted for 6 h. Blood glucose levels were measured at 0, 15, 30, 60, and 120 min after injection.

Mice with MetS were used to construct an ischemic button adhesion model. Briefly, the abdominal wall skin of the mice was prepared for disinfection after anesthesia, and a 2–3 cm incision was made along the midline of the abdomen. The hemostat gently clamped the wall of the left upper abdomen to ≈5 mm, and the bottom was ligated twice using a 5‐0 silk thread. A second ischemic button of the same size was placed in the left lower abdomen. The abdominal organs were gently rubbed ≈20 times with sterile dry gauze. The NCD+Model and HFD+Model groups were treated with 0.25 mL of N.S, and HFD+Model+hyaluronic acid (HA) and HFD+Model+ODE–ME groups were treated with 0.25 mL of HA hydrogel and 0.25 mL of ODE–ME‐15wt.% hydrogel, respectively. The incision was sutured using a 4‐0 silk thread, and the mice were allowed to recover from anesthesia on a thermal blanket. Blood glucose levels were measured on postoperative days 0, 1, 3, 5, and 7. The mice were euthanized seven days after surgery. Recovery of the incision on the abdominal wall and adhesions in the abdominal cavity were observed. Photographs were taken, and adhesions were scored. The scoring criteria are presented in Table S1 (Supporting Information).^[^ ^21^ ^]^

### H&E Staining, Masson Staining, and IHC Staining

The organs, adhesions, and intestinal and abdominal wall tissues of each group in the above models were collected. Subsequently, they were combined with 4% paraformaldehyde for 24 h, dehydrated using gradient ethanol, embedded in paraffin, and cut into sections. After dewaxing and hydration, the sections were stained with hematoxylin and eosin (H&E) solution and hematoxylin‐Ponceau red acid fuchsin‐acid aniline blue solution. After mounting with neutral gum, slides were observed under a microscope.

### Enzyme‐Linked Immunosorbent Assay Analysis

Mouse interleukin (IL)−6, tumor necrosis factor‐alpha (TNF‐α), IL‐1β, plasminogen activator inhibitor (PAI)−1, and tissue plasminogen activator (t‐PA) enzyme‐linked immunosorbent assay (ELISA) kits were purchased from the Jiangsu Jingmei Biological Technology Co., Ltd (http://www.jsjmsw.com/, Yancheng, China). Blood samples of mice from each group were collected at predetermined time points. Serum was extracted and detection reagents were added to microtiter plates and incubated for different durations according to the manufacturer's instructions. The absorbance of the plates was measured at 450 nm using a multifunctional microplate reader.

### Statistical Analysis

OriginPro 2019 and GraphPad Prism 8.4.0 software packages were used for statistical analyses and data plotting. All data are presented as mean ± standard deviation (SD). Brown–Forsythe and Bartlett tests for homogeneity of variance were performed. An ordinary one‐way analysis of variance (ANOVA) was used to compare statistical significance among multiple groups. An unpaired two‐tailed *t*‐test was used to compare the statistical significance between two groups.

## Conflict of Interest

The authors declare no conflict of interest.

## Author Contributions

X.L. and X.S. contributed equally to this work. X.L. and X.S. participated in the conception and design of the study. X.L., X.S., Z.Z., L.L., S.Y., M.C., and C.L. carried out the experiments. X.L. and X.S. acquired and analyzed the data. X.L. and X.S. drafted the manuscript. G.H., Y.Z., C.L., and X.L. contributed to the design of the study. G.H. and Y.Z. revised the manuscript. All authors have approved the final version of the manuscript.

## Supporting information

Supporting Information

## Data Availability

The data that support the findings of this study are available from the corresponding author upon reasonable request.
