# Supplementary material for: Multifunctional Oxidized Dextran–Metformin as a Tissue‐Adhesive Hydrogel to Prevent Postoperative Peritoneal Adhesions in Patients with Metabolic Syndrome
Source: Adv Sci (Weinh). 2023 Oct 16;10(33):2303767. doi: 10.1002/advs.202303767 (PMC10667813; doi:10.1002/advs.202303767)
Supplement: Supplementary file 1 — Supporting Information [file ADVS-10-2303767-s001.pdf]

ADVANCED SCIENCE
Open Access

Supporting Information

for *Adv. Sci.*, DOI 10.1002/advs.202303767

Multifunctional Oxidized Dextran–Metformin as a Tissue-Adhesive Hydrogel to Prevent Postoperative Peritoneal Adhesions in Patients with Metabolic Syndrome

Xi Liu, Xianwen Song, Zequn Zhang, Shutong Yang, Liang Li, Changwei Lin, Miao Chen, Chuntai Liu, Xiaorong Li, Yi Zhang and Gui Hu**

Table S1. Scoring system of mouse ischemic button model

Score	Description
0	No visible adhesion to the ischemic button, with limited mesothelial thickening on the button.
1	A string adhesion to the ischemic button.
2	Direct attachment of one area of an organ to the ischemic button, the adhesion contact itself was light and usually involved contact between the peritoneum and an abdominal organ.
3	Direct attachment of two non-continuous areas of one organ or two areas of two organs to the ischemic button, usually between the peritoneum and two abdominal organs or two distinct, noncontinuous areas of a single organ.
4	Direct attachment of three or more non-continuous areas to the ischemic button, usually between the peritoneum and three abdominal organs, or multiple separate areas of one or two organs.
5	Full compaction/encapsulation of the abdominal organs, most organs were adhered to the peritoneum as well as to each other as a single, rigid mass.

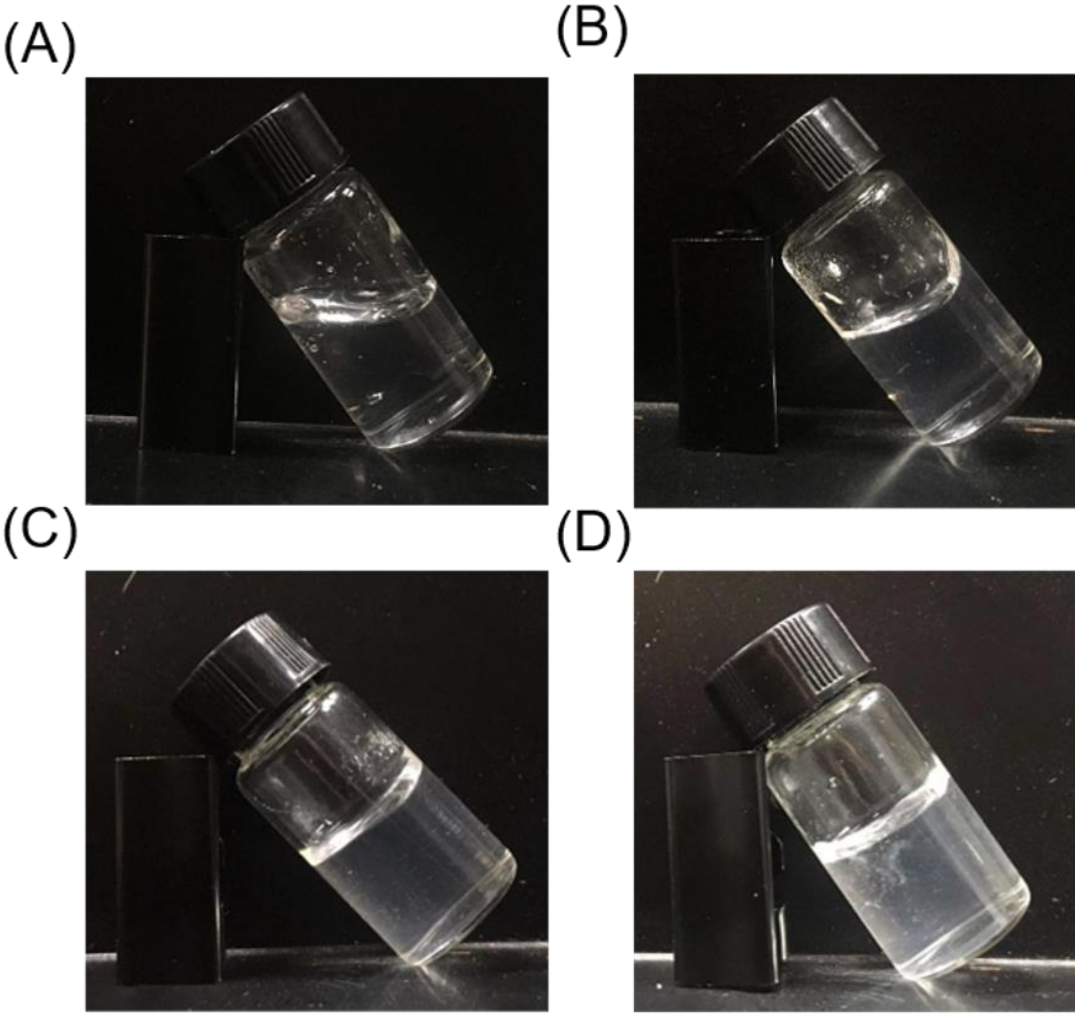

Figure S1. Pictures of ODE-ME in aqueous solution at concentrations of (A) 5 wt%, (B) 10 wt%, (C) 15 wt%, and (D) 20 wt%.

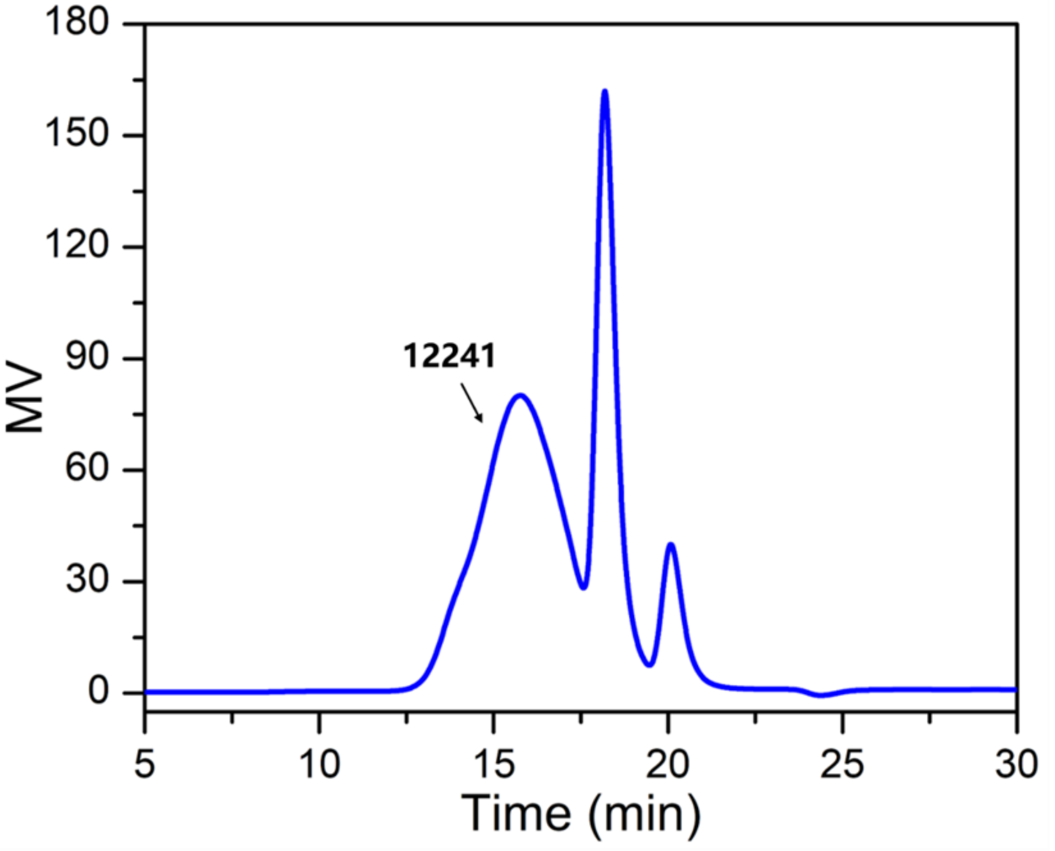

Broad Unknown Relative Peak Table

	Distribution Name	Mn (Daltons)	Mw (Daltons)	MP (Daltons)	Mz (Daltons)	Mz+1 (Daltons)	Polydispersity	Mz/Mw	Mz+1/Mw
1		9757	21133	12241	44466	70071	2.165924	2.104121	3.315759

Figure S2. Gel Permeation Chromatography (GPC) chromatogramof ODE-ME hydrogel.

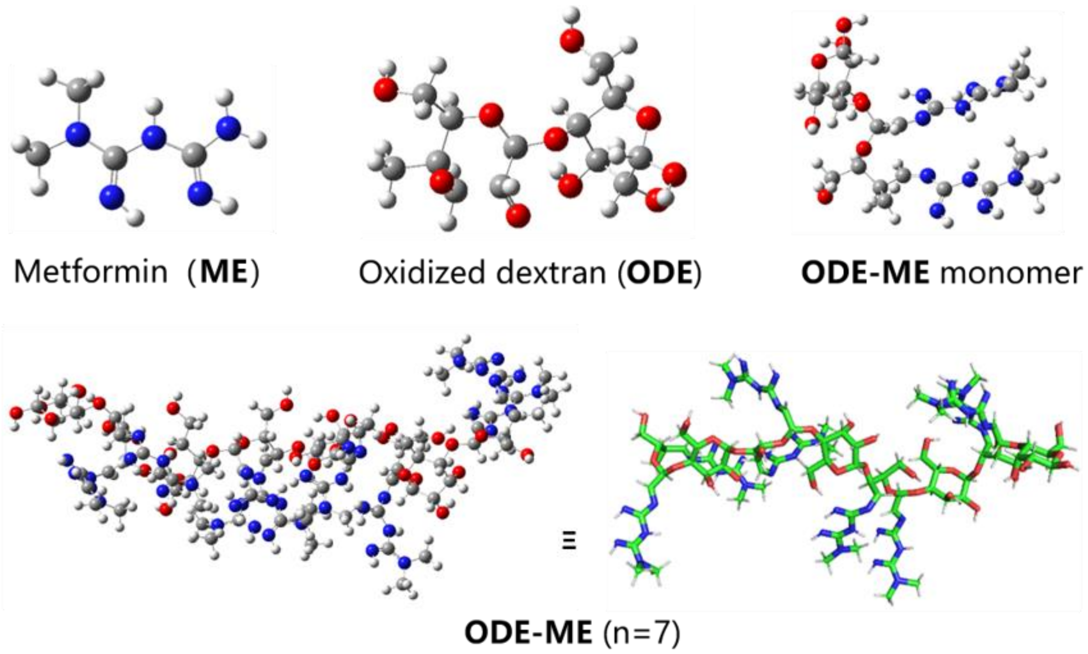

Figure S3. Molecular structure of ODE-ME molecular fragment.

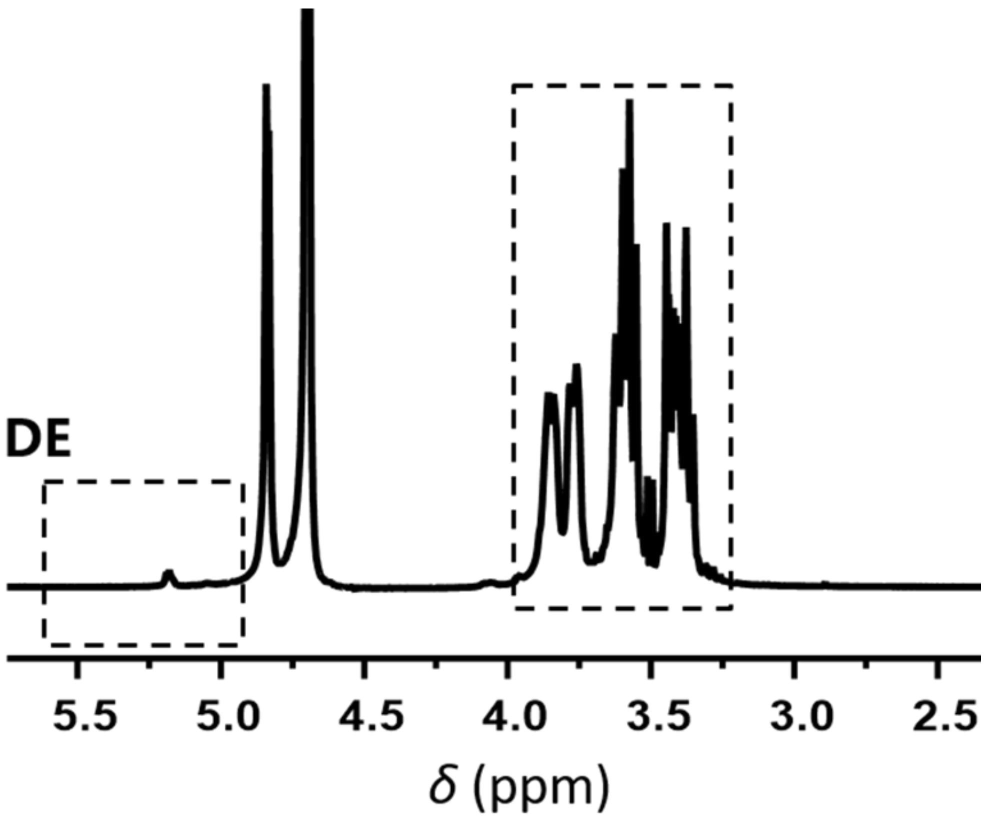

Figure S4. ^{1}H NMR spectra of the DE molecular.

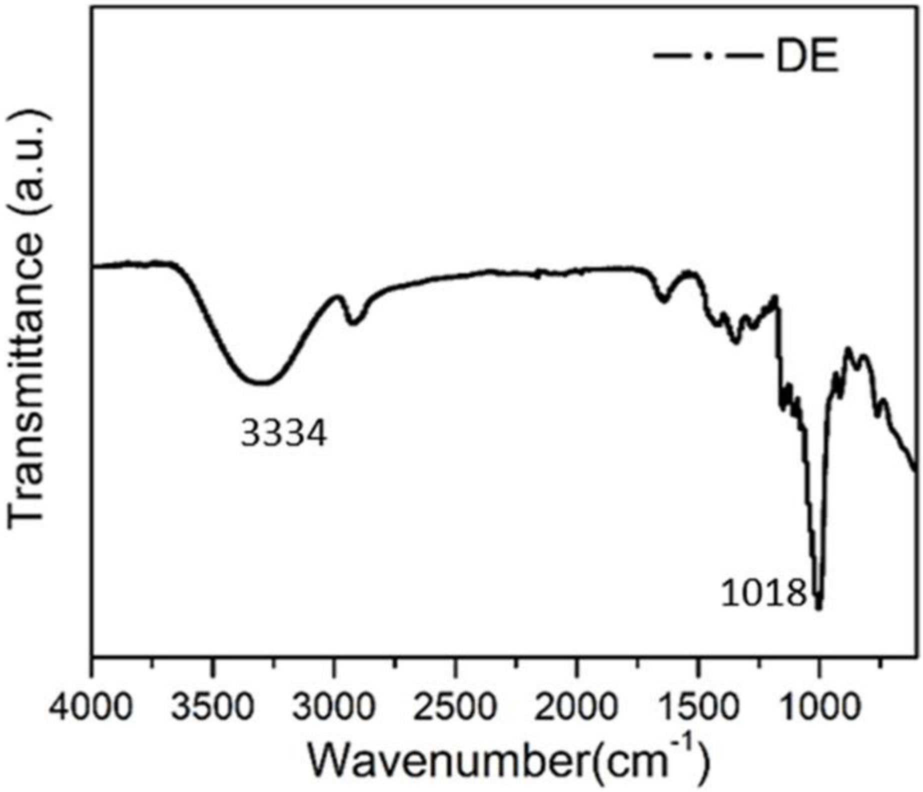

Figure S5. FT-IR spectra of the DE molecular.

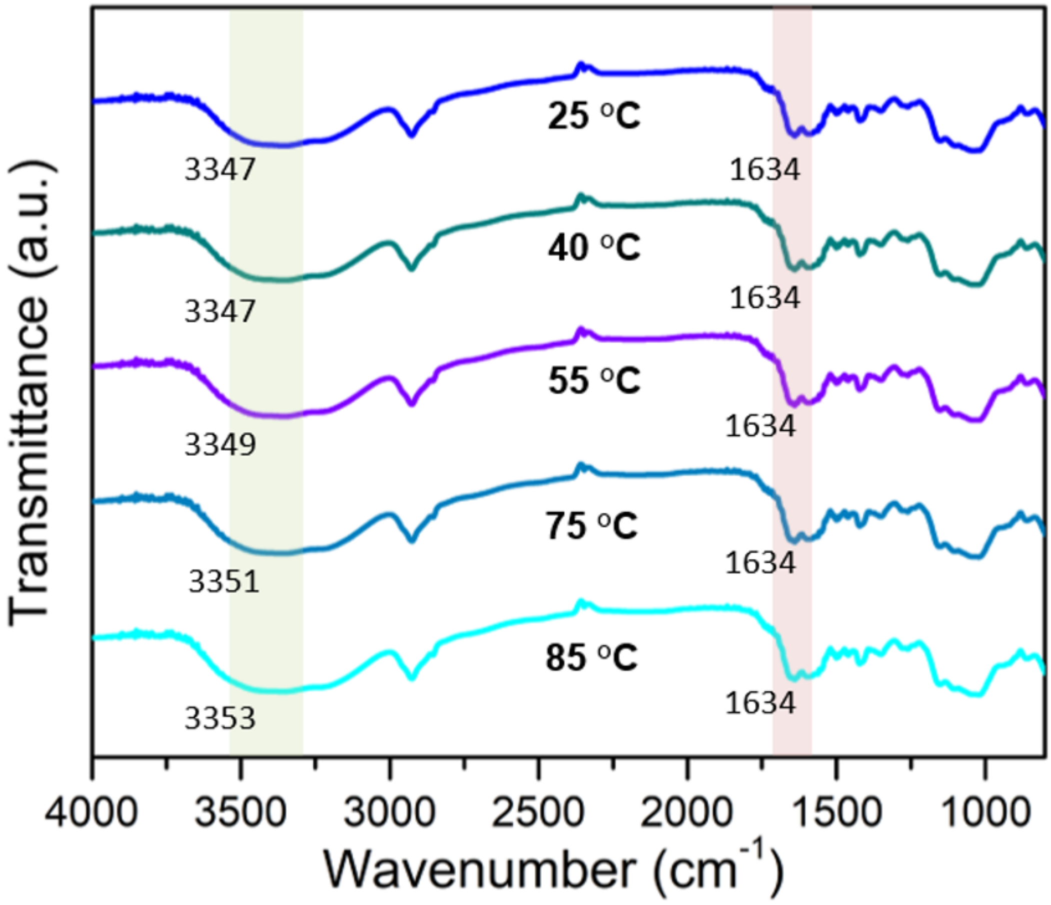

Figure S6. Variable-temperature FTIR spectroscopy of ODE-DE samples.

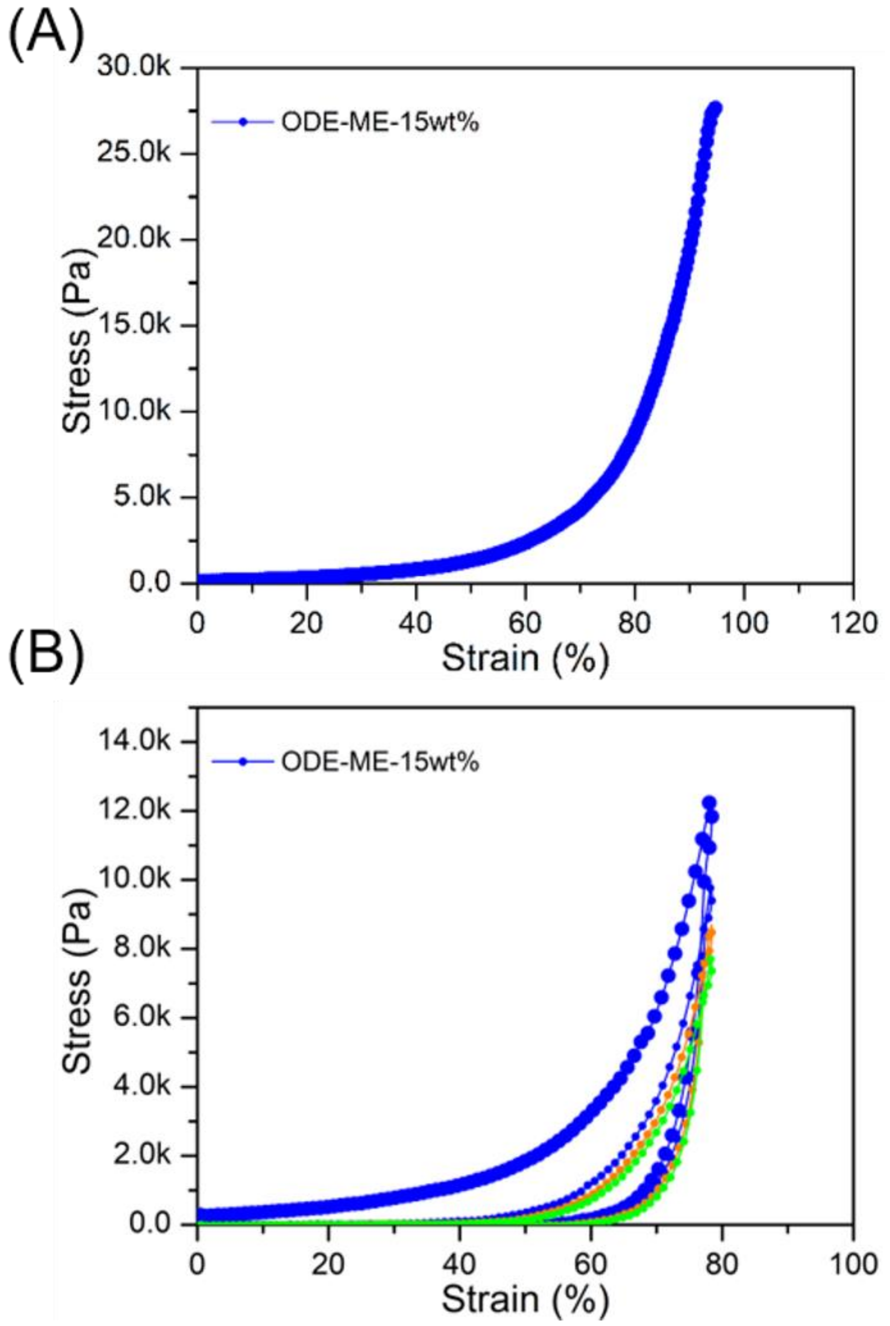

Figure S7. (A) Stress-strain curves and (B) continuous loading-unloading tests of ODE-DE -15wt% hydrogels in compression.

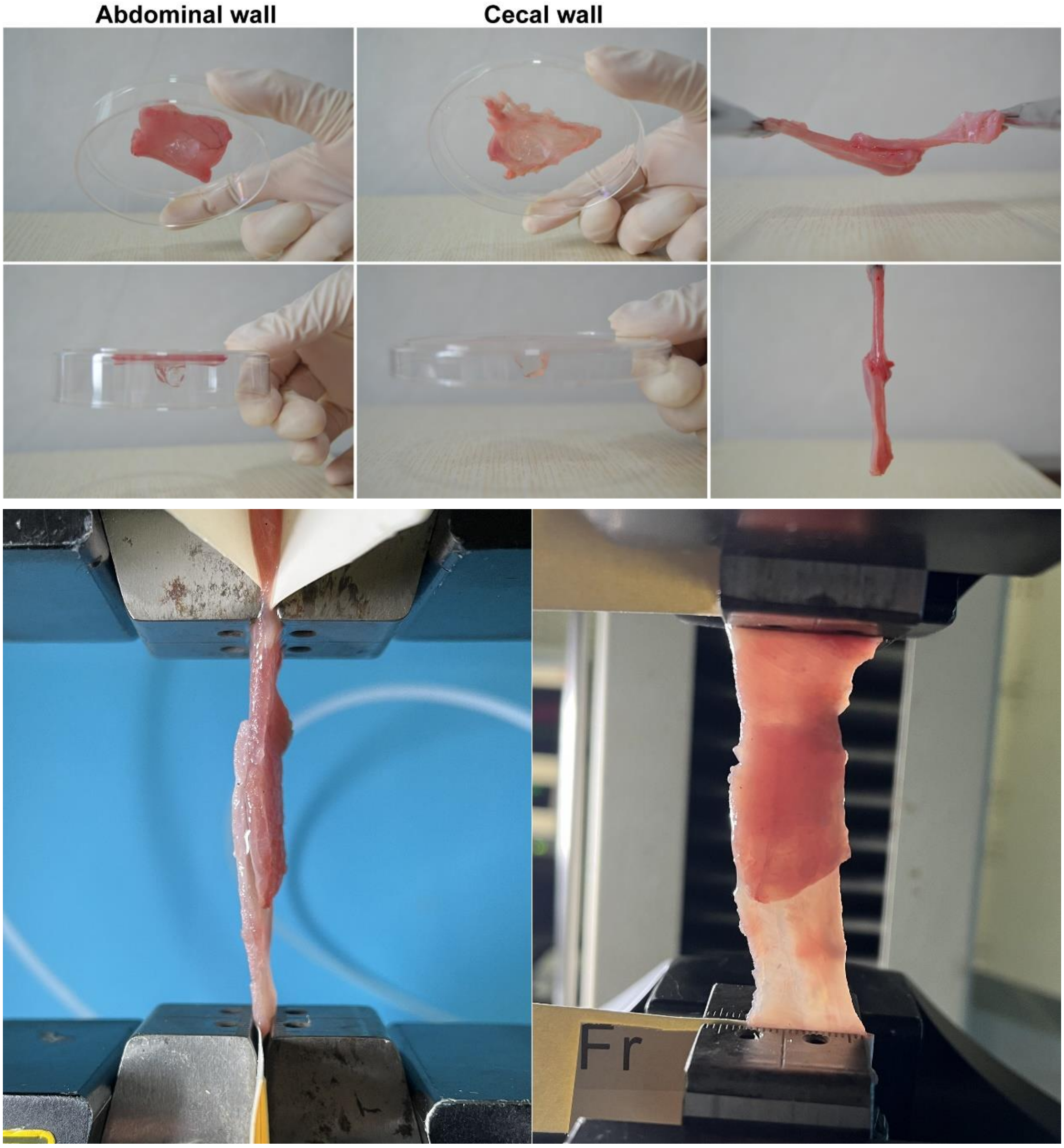

Figure S8. Tissue-adhesive and lap shear test of ODE-ME hydrogels.

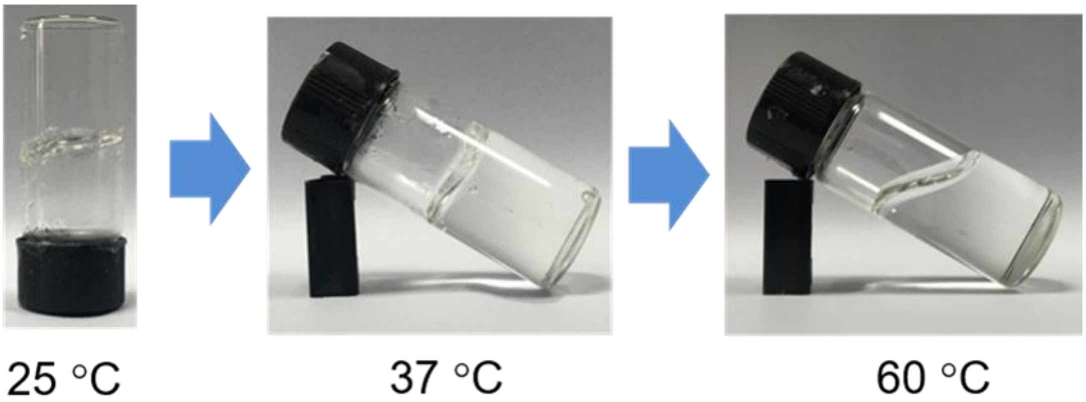

Figure S9. ODE-ME hydrogels at different temperatures.

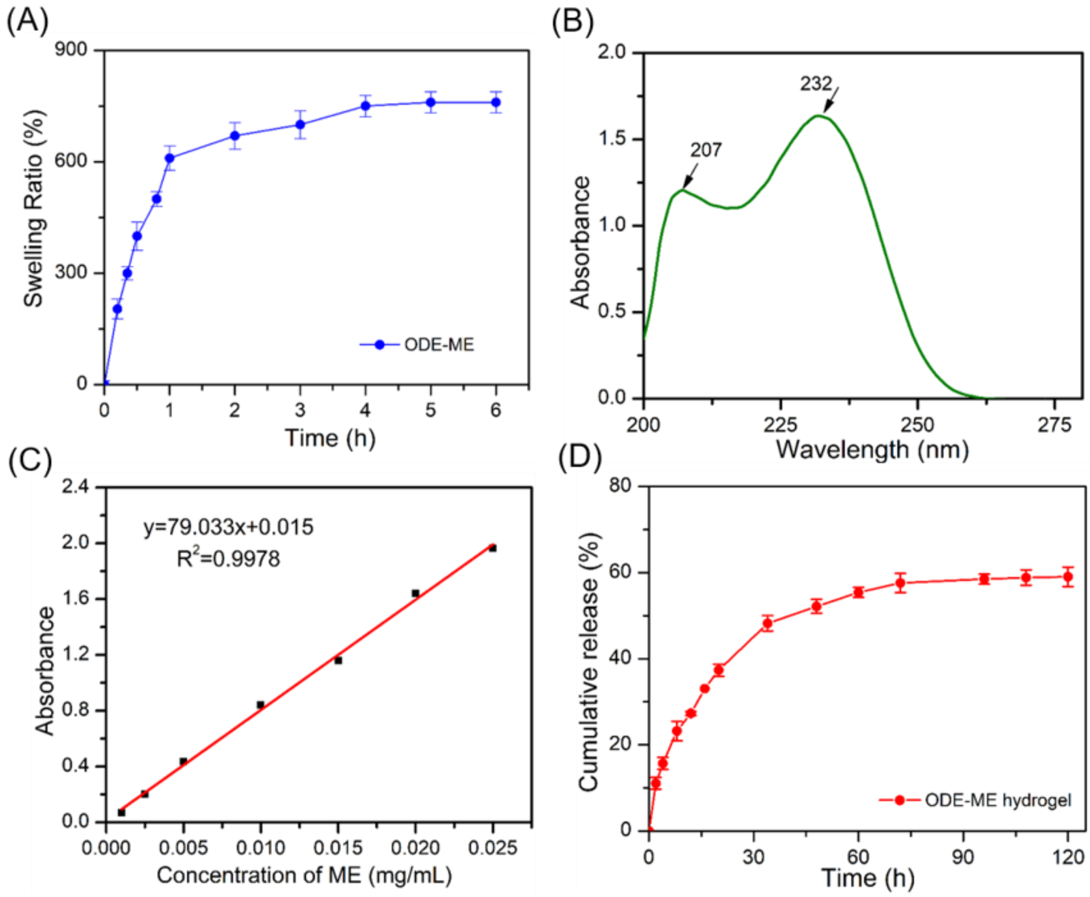

Figure S10. (A) Swelling test of hydrogels. (B) UV-absorption pattern of metformin. (C) Standard curve of ME, as constructed using a series of known ME concentrations in PBS solution. (D) Releasing behavior of the ODE-ME hydrogel.

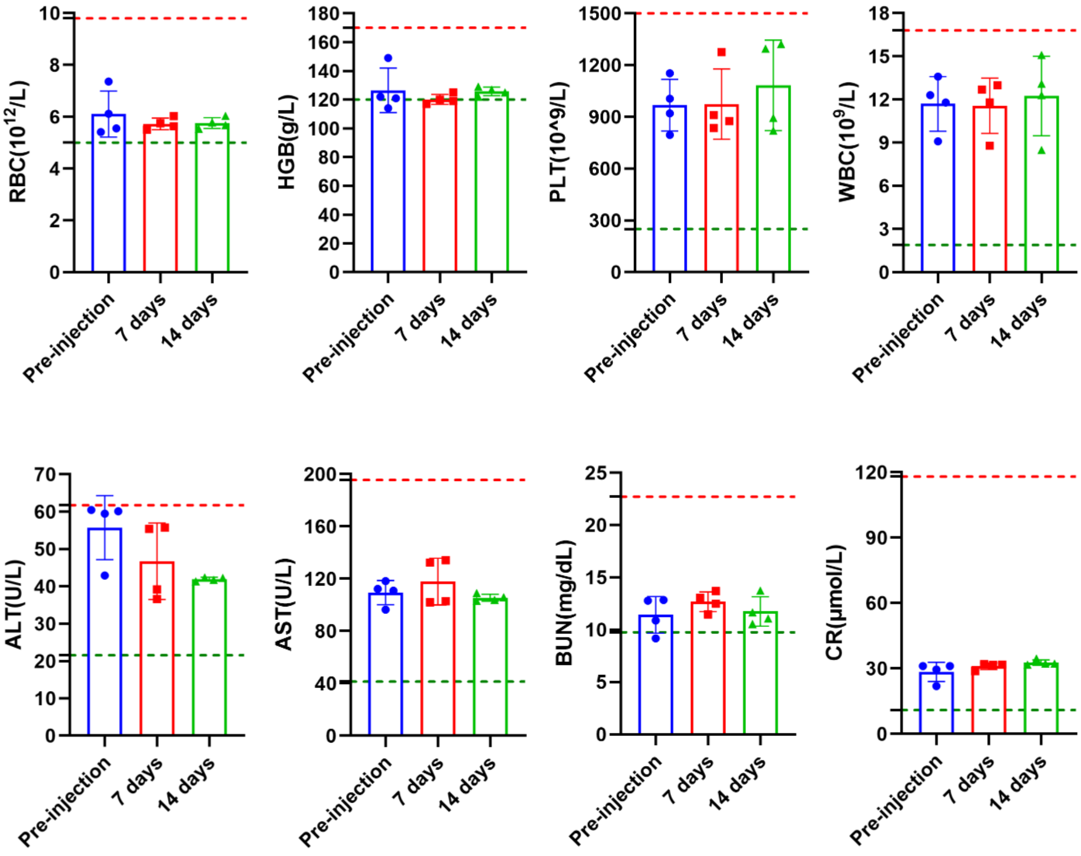

Figure S11. Blood biochemical examination and routine examination results of rats after intraperitoneal injection of ODE-ME-15wt% hydrogels (n = 4). The red dotted lines represent the upper limit of normal physiological values. The green dotted lines represent the lower limit of normal physiological values. WBC: White Blood Bells, PLT: Platelets, RBC: Red Blood Cells, HGB: Hemoglobin, ALT: Alanine aminotransferase, AST: Aspartate aminotransferase, BUN: Blood Urea Nitrogen, CR: Creatinine.

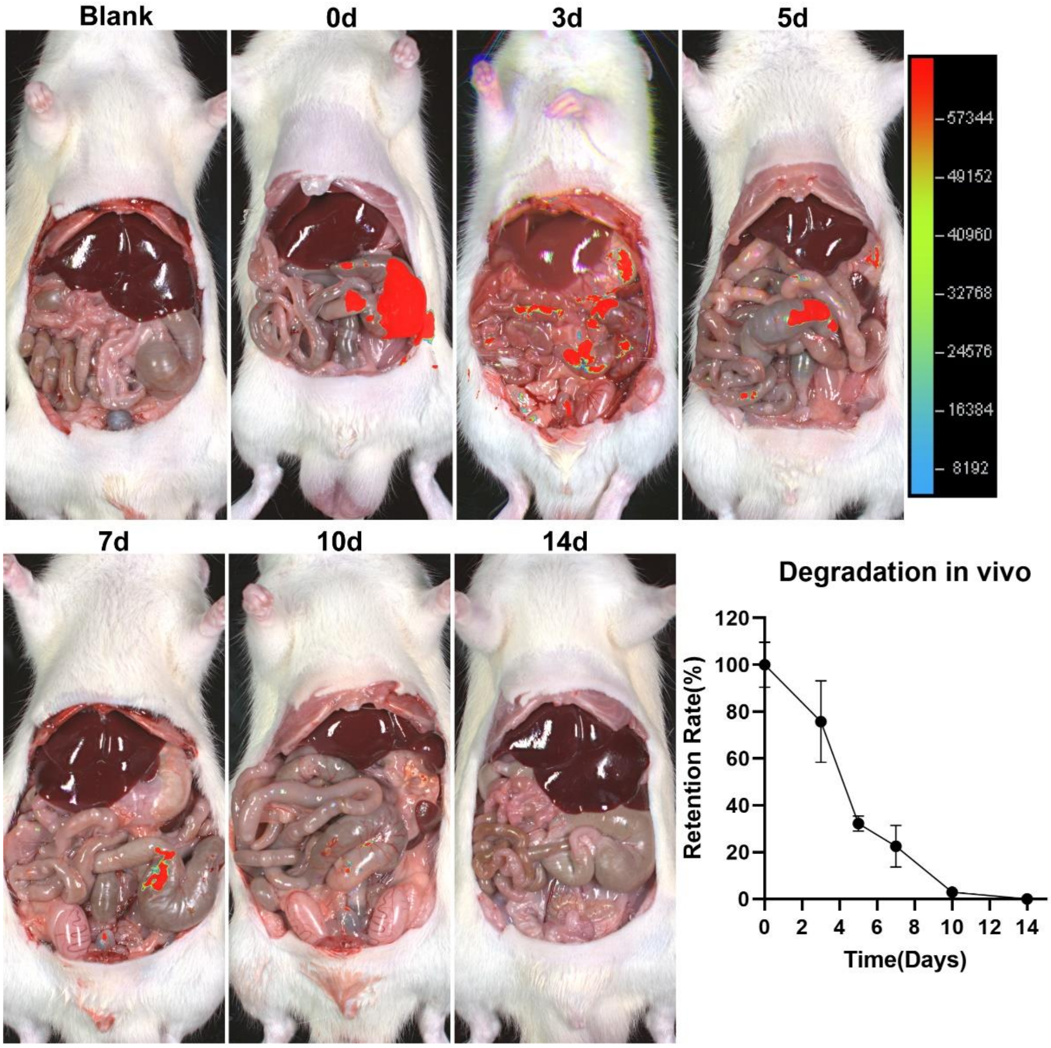

Figure S12. Representative images of *in vivo* imaging at different time points after FITC-labeled ODE-ME-15wt% hydrogel was injected into the abdominal cavity and Quantitative statistical analysis of the degradation rate (n = 3).

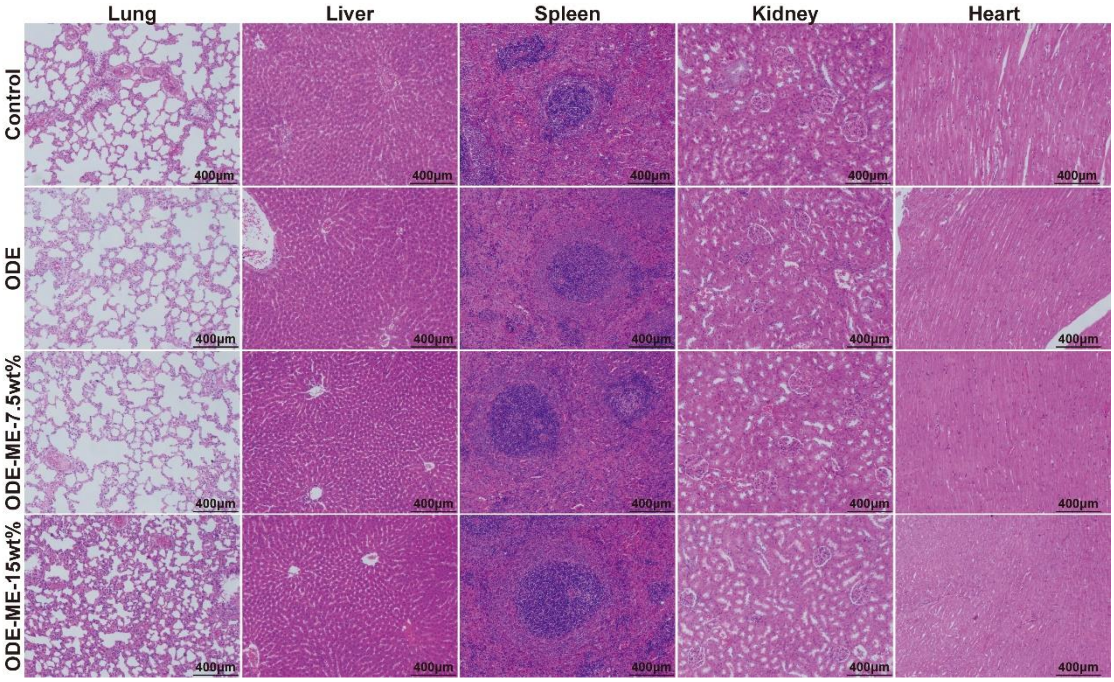

Figure S13. H&E staining results to evaluate the effects of ODE-ME hydrogels on heart, liver, spleen, lung, and kidney.

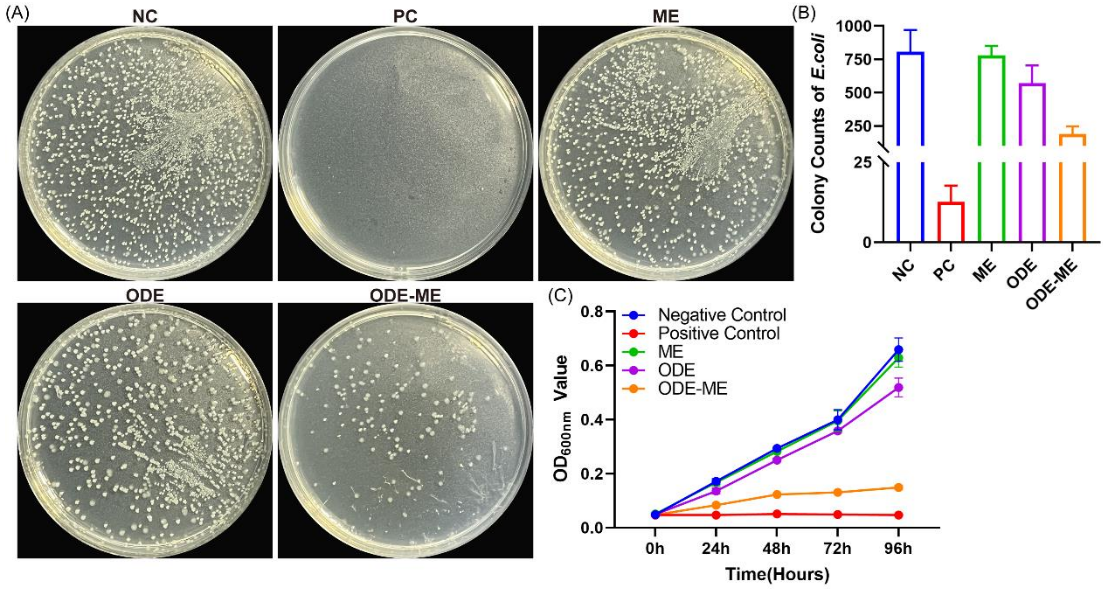

Figure S14. (A) Representative display pictures and (B) quantitative statistical data of ODE-ME hydrogels anti-*E.coli* test (n = 3). (C) Time-dependent antibacterial test toward *E.coli* (n = 3).

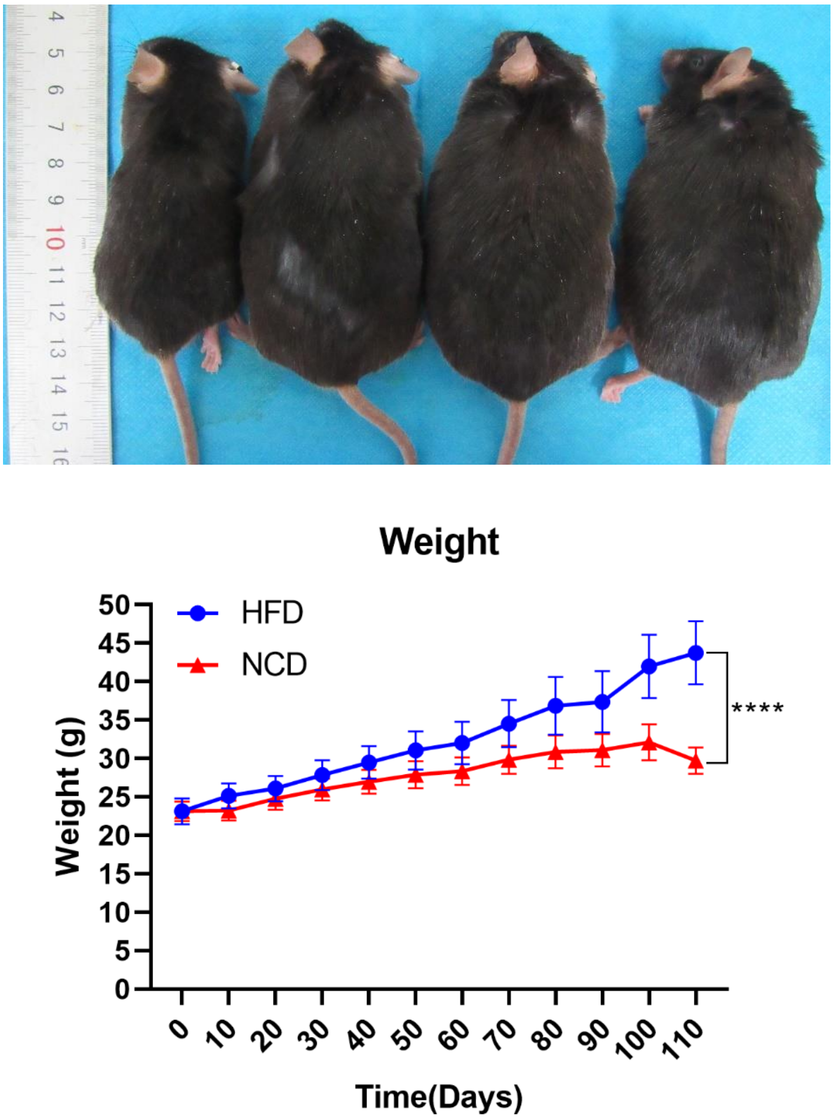

Figure S15. General photographs of mice after NCD and HFD feeding and Body weight changes.

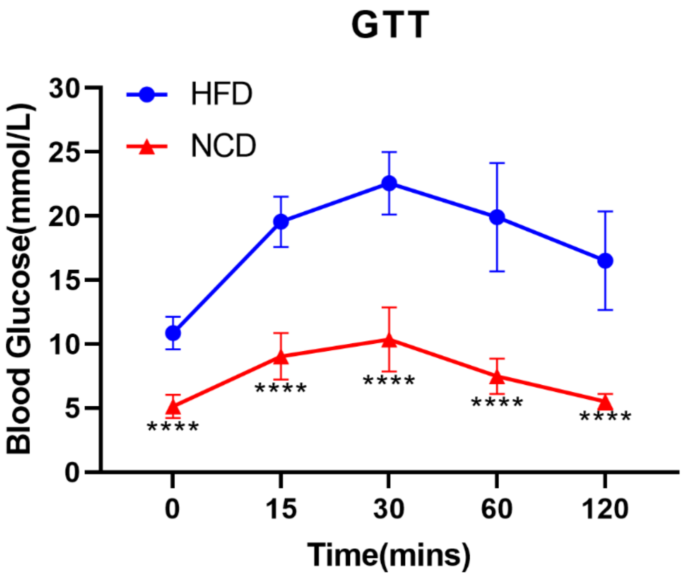

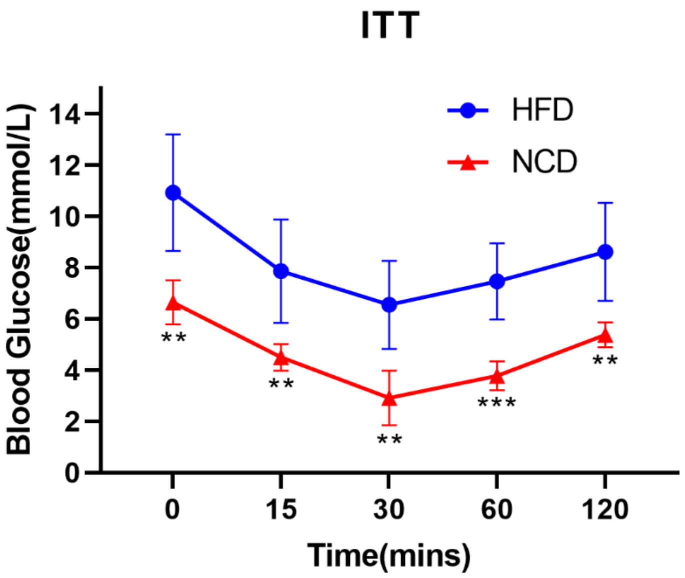

Figure S16. Glucose tolerance tests (GTTs) and insulin tolerance tests (ITTs) results of mice fed with NCD and HFD (n = 6).

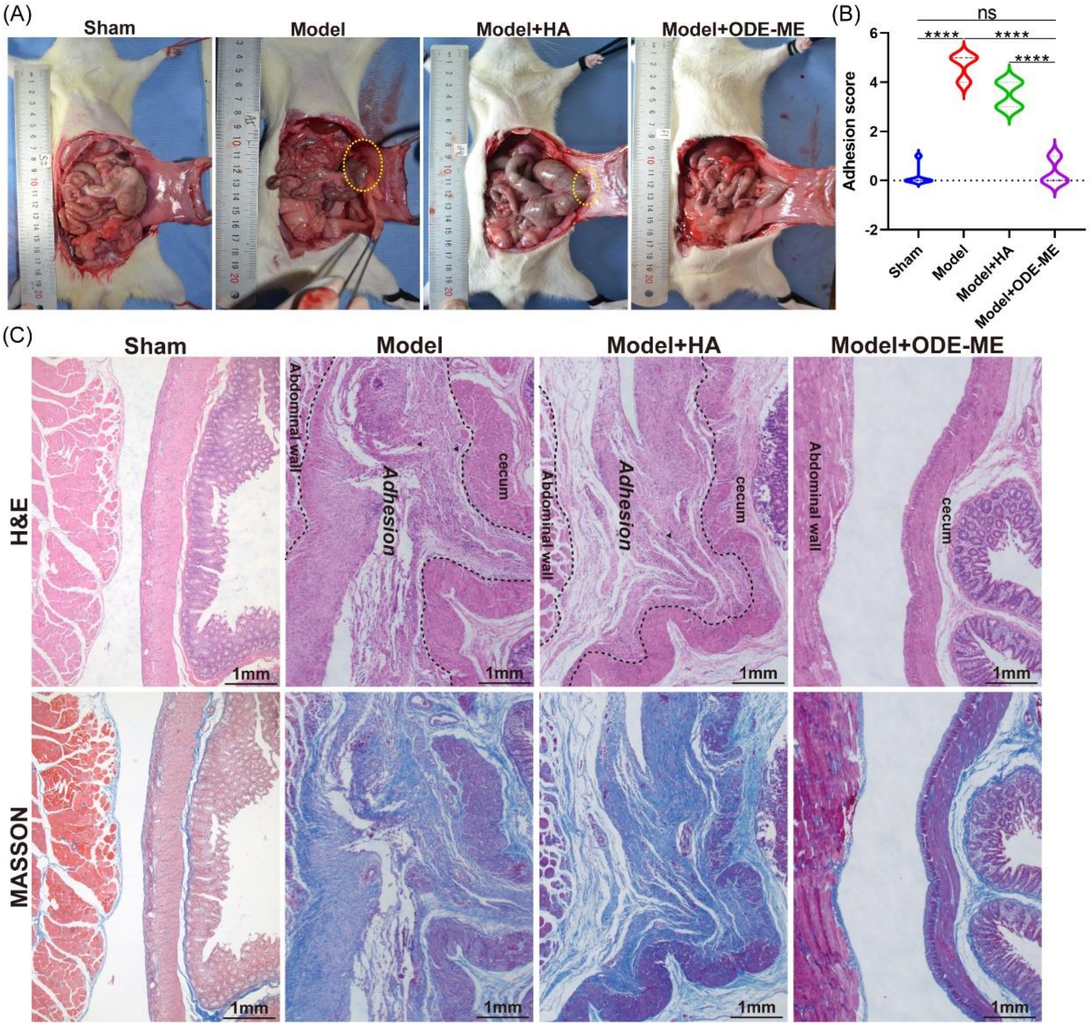

Figure S17. Prevention of cecum abrasion-sidewall defect by the ODE-ME hydrogel in the adhesion model. (A) Representative photographs of postoperative peritoneal adhesions on day 7 and (B) adhesion scores of different groups (n = 6). (C) Results of representative H&E staining and Masson staining of each group on day 7 after the first surgery.

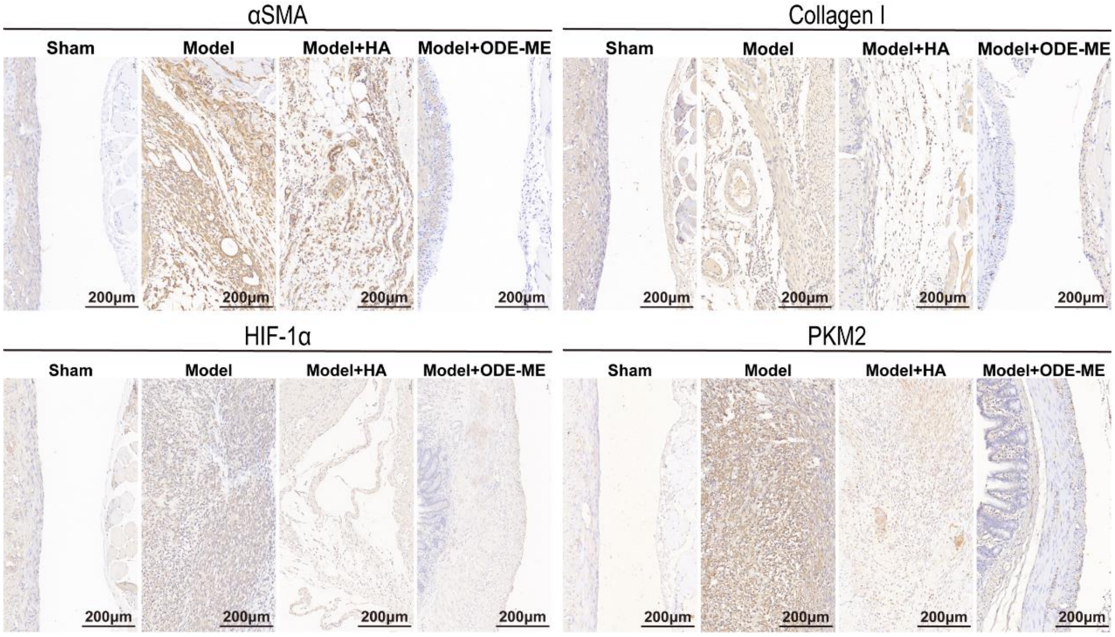

Figure S18. Representative images of IHC staining of αSMA, Collagen I, HIF1α and PKM2 on day 7 after the first surgery.
